# β-Patchoulene Ameliorates Water Transport and the Mucus Barrier in 5-Fluorouracil-Induced Intestinal Mucositis Rats *via* the cAMP/PKA/CREB Signaling Pathway

**DOI:** 10.3389/fphar.2021.689491

**Published:** 2021-08-25

**Authors:** Jiazhen Wu, Yuxuan Gan, Huijuan Luo, Nan Xu, Liping Chen, Mengyao Li, Fengkun Guan, Ziren Su, Zhixiu Lin, Jianhui Xie, Yuhong Liu

**Affiliations:** ^1^ School of Pharmaceutical Sciences, Guangzhou University of Chinese Medicine, Guangzhou, China; ^2^ The Second Clinical Medical College, Guangzhou University of Chinese Medicine, Guangzhou, China; ^3^ Faculty of Health Sciences, University of Macau, Macao, China; ^4^ Department of Basic Research, Dongguan Institute of Guangzhou University of Chinese Medicine, Dongguan, China; ^5^ State Key Laboratory of Dampness Syndrome of Chinese Medicine, The Second Affiliated Hospital of Guangzhou University of Chinese Medicine, Guangzhou, China; ^6^ Guangdong Provincial Key Laboratory of Clinical Research on Traditional Chinese Medicine Syndrome, The Second Affiliated Hospital of Guangzhou University of Chinese Medicine, Guangzhou, China

**Keywords:** intestinal mucositis, *β*-patchoulene, water transport, cAMP/PKA/CREB pathway, 5-fluorouracil

## Abstract

Intestinal mucositis (IM) is the main side effect observed in patients who receive cancer chemotherapy. The characteristics of ulceration, vomiting, and severe diarrhea cause patients to delay or abandon further treatment, thereby aggravating their progress. Hence, IM cannot be overlooked. *β*-patchoulene (*β*-PAE) is an active ingredient isolated from *Pogostemon cablin* (Blanco) Benth (Labiatae) and has shown a marked protective effect against gastrointestinal diseases in previous studies. However, whether *β*-PAE plays a positive role in IM is still unknown. Herein, we explore the effects and the underlying mechanism of *β*-PAE against 5-fluorouracil (5-FU)-induced IM in IEC-6 cells and rats. *β*-PAE significantly recovered cell viability, upregulated the IM-induced rat body weight and food intake and improved the pathological diarrhea symptoms. Aquaporin is critical for regulating water fluid homeostasis, and its abnormal expression was associated with pathological diarrhea in IM. *β*-PAE displayed an outstanding effect in inhibiting aquaporin 3 (AQP3) *via* the cAMP/protein kinase A (PKA)/cAMP-response element-binding protein (CREB) signaling pathway. Besides, inflammation-induced mucus barrier injury deteriorated water transport and aggravated diarrhea in IM-induced rats. *β*-PAE’s effect on suppressing inflammation and recovering the mucus barrier strengthened its regulation of water transport and thus alleviated diarrhea in IM-induced rats. In sum, *β*-PAE improved IM in rats mainly by improving water transport and the mucus barrier, and these effects were correlated with its function on inhibiting the cAMP/PKA/CREB signaling pathway.

## Introduction

Chemotherapy is an effective method for curing cancer, though it is inevitably associated with the painful side effect of mucositis. It is a major side effect for tumor patients who receive cancer chemotherapy and includes oral mucositis and intestinal mucositis (Van Sebille et al., 2015). 5-fluorouracil (5-FU) is a first-line clinical antineoplastic drug used over the past 30 years, but its frequent adverse effect, intestinal mucositis (IM), contributes to suboptimal treatment outcomes in patients (Longley et al., 2003; Ribeiro et al., 2016). IM induces complex inflammatory damage to the mucosa in the gastrointestinal tract (GIT) and leads to patient’s inability to eat, weight loss, and local infection (Sharma et al., 2005). Clinically, IM patients exhibit a series of uncomfortable reactions in the GIT, such as ulceration, vomiting, and diarrhea (Touchefeu et al., 2014). These phenotypes exacerbate cancer development and finally cause death (Pulito et al., 2020). Loperamide (LP) is a recommended drug in current guidelines that is used for the treatment of chemotherapy-induced diarrhea (Stein et al., 2010). However, more treatment strategies should be explored to improve IM. Currently, an increasing number of studies have indicated that IM development involved mucus barrier injury, intestinal inflammation, and water transport dysfunction (Groschwitz and Hogan, 2009; Al-Dasooqi et al., 2013; Ikarashi et al., 2016). Improving these defects would be a potential treatment strategy for ameliorating IM.

*Pogostemon cablin* (Blanco) Benth (Labiatae) is a well-known traditional healthy food and medicinal herb used in Asian countries, and its essential oil (patchouli oil) protects the intestinal barrier by reducing inflammation and alleviates diarrhea by restoring intestinal water absorption in IM-induced rats (Hou and Jiang, 2013; Gan et al., 2020). *β*-patchoulene (*β*-PAE) is the main active ingredient extracted from patchouli oil, but whether it attenuates diarrhea and inflammation in IM-induced rats is still unknown. Diarrhea is a common symptom in IM patients and is associated with water transport activity, which is regulated by aquaporins (AQPS). Reduced AQP3 expression was most significant after patchouli oil treatment in IM-induced rats. Therefore, we evaluated the function of *β*-PAE on AQP3 expression and the underlying pathways. Furthermore, inflammation-induced mucus barrier injury is also involved in disordered water transport, which exacerbates diarrhea symptoms. *β*-PAE exerts an anti-inflammatory effect in ulcerative colitis and gastric ulcer (Wu et al., 2018; Liu et al., 2020). Decreasing the inflammatory response to achieve water transport homeostasis may represent a potential bypass mechanism of *β*-PAE to improve IM, which was also evaluated in this study.

Apart from *β*-PAE, patchouli alcohol (PA) is also a non-negligible ingredient in patchouli oil because of its various bioactivities. PA also protected rats against 5-FU-induced IM by improving diarrhea and the intestinal barrier (Wu et al., 2020), but its effect on water transport is currently unknown; hence, the objectives of the present study.

## Materials and Methods

### Materials

**FIGURE 1:**
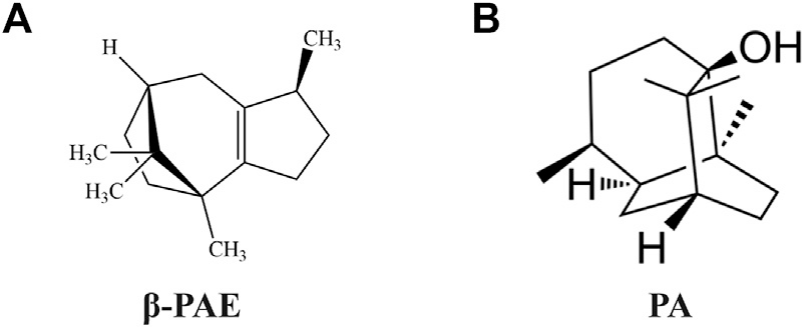
Chemical structure formula. **(A)** *β*-PAE; **(B)** PA.

β-PAE (Figure 1A, PubChem CID: 101731) and PA (Figure 1B, PubChem CID: 10955174) were isolated from *P. cablin* according to our previous methods (Su et al., 2014; Zhang et al., 2016). 5-fluorouracil (5-FU, WXBC5949V) was purchased from Sigma-Aldrich Co., Ltd. (Shanghai, China). Loperamide hydrochloride (LP) was purchased from Janssen Pharmaceutical Co., Ltd. (Xi’an, China). 8-Bromo-cAMP (8-Br-cAMP) was purchased from Selleck Chemicals Co., Ltd. (Texas, United States). Primary antibodies against rat Occludin, Claudin-1, p-p65, p65, vasoactive intestinal polypeptide (VIP), vasoactive intestinal polypeptide receptor 2 (VIPR2), protein kinase A (PKA), aquaporin 3 (AQP3), cAMP-response element-binding protein (CREB), p-CREB, extracellular regulated protein kinases (ERK), p-ERK, p38, p-p38, MEK1/2, p-MEK1/2, P300/CBP, and the secondary antibodies were obtained from Affinity Biosciences Co., Ltd. (OH, United States). Cyclic adenosine monophosphate (cAMP), p-MSK1, and *β*-actin were obtained from Abcam Co., Ltd. (Cambridge, United Kingdom). MSK1 was obtained from Proteintech Group, Inc. (Wuhan, China). All other chemicals or solvents were of analytical grade.

### Cell Culture

Rat intestinal epithelial cell line IEC-6 was gifted from Blue Biotechnology Development Co., Ltd. (Shanghai, China). IEC-6 cells were cultured in completed high glucose DMEM medium supplemented with 10% FBS (v/v) and 1% penicillin/streptomycin (v/v) at 37°C, 5% CO_2_ in a humidified incubator (Thermo Fisher, MA, United States) (Jeffrey et al., 2018).

### Cell Viability Evaluation

IEC-6 cells in the logarithmic (log) growth phase were seeded in 96-well plates at a density of 6,500 cells per well and treated with 5-FU (0.1, 1, 10, 20, 40, 60, 80, 100, and 200 μM) or *β*-PAE (0.1, 1, 10, 20, 40, 60, 80, and 160 μM) for 24 and 48 h. Next, 10 μL of the CCK-8 solution was added to each well of the plate and then the culture plate was placed in an incubator at 37°C for 2 h. The absorbance of each well was measured at 450 nm using a microplate reader.

### Treatment of IEC-6 Cells With *β*-Patchoulene

The CCK-8 assay ensured an optimal dose of 5-FU and *β*-PAE when cells were treated for 24 h and the results obtained to determine the IC50 were used in the subsequent experiments.

IEC-6 cells in the log growth phase were seeded into 6-well plates at a density of 2 × 10^5^ cells/mL per well over 24 h and induced by DMSO or 100 μM 8-Br-cAMP for 24 h. Afterward, treatment groups were exposed to *β*-PAE with or without 5-FU for 24 h. At the end of the study period, the above cell samples were collected for protein expression measurement by western blotting.

### Animals

All animal protocols of this study were performed following the guidelines of the Animal Experimental Ethics Committee of Guangzhou University of Chinese Medicine (No. 20190322003). Male Sprague Dawley rats (180–220 g, SYXK (YUE) 2013-0085) were reproduced from Guangdong Medical Laboratory Animal Center (Foshan, China) and housed at experimental animal central of Guangzhou University of Chinese Medicine. All experimental rats were raised at cages at controlled room temperature (23 ± 2°C) and humidity (50 ± 10%) with a half-day light/dark cycle. Additionally, the rats were allowed free access to food and clean water throughout the experimental procedure.

### Induction of Intestinal Mucositis

The method for making rats intestinal mucositis was slightly modified (Gan et al., 2020). In this part, two animal studies were performed: (1) evaluation of the potency and dose dependence of *β*-PAE. Rats were randomly assigned to six treatment groups (*n* = 8): control group, 5-FU group (35 mg/kg), LP group (10 mg/kg), and *β*-PAE groups (10, 20, and 40 mg/kg); and (2) comparative study between *β*-PAE and PA. Rats were randomly assigned to four treatment groups (*n* = 6): control group, 5-FU group (35 mg/kg), *β*-PAE group (40 mg/kg), and PA group (40 mg/kg). The control rats were intraperitoneally injected with normal saline only, while other groups received intraperitoneal injections of 5-FU (35 mg/kg, dissolved in normal saline) daily from day 1 to day 4. Simultaneously, rats received LP or different doses of *β*-PAE by oral gavage according to their respective treatment group, while control rats received normal saline by oral gavage during the experimental procedure. Body characteristics including body weight, food intake, and diarrhea scores (Table 1) were recorded each day (Bowen et al., 2007). At the end of treatment, rats were euthanized by using pentobarbital sodium (i.p., 60 mg/kg) (Garcia-Pedraza et al., 2015), small intestine (ileum) tissues were collected, and blood samples were collected from the abdominal aorta for biochemical analysis.

**TABLE 1:** Diarrhea scores criteria.

Grade	Description
0	No diarrhoea
1	Mild diarrhea (Slightly wet, loose feces)
2	Moderate diarrhea (Wet, amorphous feces)
3	Severe diarrhea (Watery stool, staining over perianal region)

### Histopathological Assessment

The small intestine tissue sections were obtained from paraffin-embedded samples and were stained with hematoxylin and eosin (H&E) for histopathological evaluation. Histopathological scores were calculated by three peer reviewers according to a previous study with a slight modification (Tang et al., 2017), including the reduced villus height, swollen crypt, and inflammatory cell infiltration. Each parameter was examined separately using histopathological scores ranging from 0 (no damage) to 3 (severe damage).

### Immunohistochemical Analysis

The small intestine tissue sections were washed with phosphate-buffered saline, blocked with 10% bovine serum albumin (BSA), and incubated with primary anti-MUC2 mucin antibodies (dilution 1:100) overnight. Then sections were incubated with goat anti-rabbit secondary antibody labeled by horseradish peroxidase. Subsequently, the slides were stained with 3,3′-diaminobenzidine (DAB) and stained with hematoxylin. Sections were observed under a light microscope and photographed. The images obtained were used to measure the integrated optical density (IOD).

### Periodic Acid-Schiff Stain Analysis

The small intestine tissue sections dewaxed to water were oxidized by 0.5% Periodate water and stained by Schiff reagent (PAS) in the darkroom. Subsequently, slides were stained with hematoxylin and then observed under a light microscope and photographed. The images obtained were used to measure the number of goblet cells.

### Intestinal Inflammatory Cytokines and Aquaporin3 Levels Measurement by Enzyme-Linked Immunosorbent Assay

Intestinal levels of TNF-α, Interleukin (IL)-1β, IL-6, IL-10, and AQP3 were measured by ELISA kits according to the manufacturer’s instructions (MLBIO Biotechnology Co. Ltd. and Elabscience Biotechnology Co., Ltd.).

### Proteins Expression Evaluation by Western Blotting

Tissues and cells were homogenized in RIPA lysis buffer containing protease inhibitors, and then protein concentration was quantified using a BCA kit (BestBio, Shanghai, China). Equal amounts of denatured protein were separated on a 10 or 12% sodium dodecyl sulfate-polyacrylamide gels (SDS-PAGE) for electrophoresis and then were transferred to polyvinyl difluoride (PVDF) membranes. Protein-loaded PVDF membranes were immersed into 5% skim milk for 1 h, and subsequently, membranes were incubated with primary antibodies and secondary antibodies as per the manufacturer's instructions. Antibodies were diluted using 1× Tris Buffered Saline with 0.1% Tween 20 (TBST) before incubation with membranes (all primary antibodies were 1:2,000 and secondary antibodies were 1:3,000 in this study). Subsequently, the membranes were washed three times for 10 min each with TSBT. Protein expression was detected using an Electro-Chemi-Luminescence (ECL) reagent (Tanon, Shanghai, China).

### Data Analysis

Statistical analysis was performed using Statistical Product and Service Solutions (SPSS) software 26.0 (IBM Corp., Armonk, NY, United States). Data were statistically analyzed by one-way analysis of variance (parametric data) and showed as mean ± standard deviation (SD). Spearman correlations were performed to analyze the association between indexes. The significance levels were determined based on the *p*-values (*p* < 0.05 was considered statistically significant).

## Results

### β-PAE Suppressed Aquaporin3 Expression by Inactivating the Protein Kinase A/cAMP-Response Element-Binding Protein Signaling Pathway in IEC-6 Cells

**FIGURE 2:**
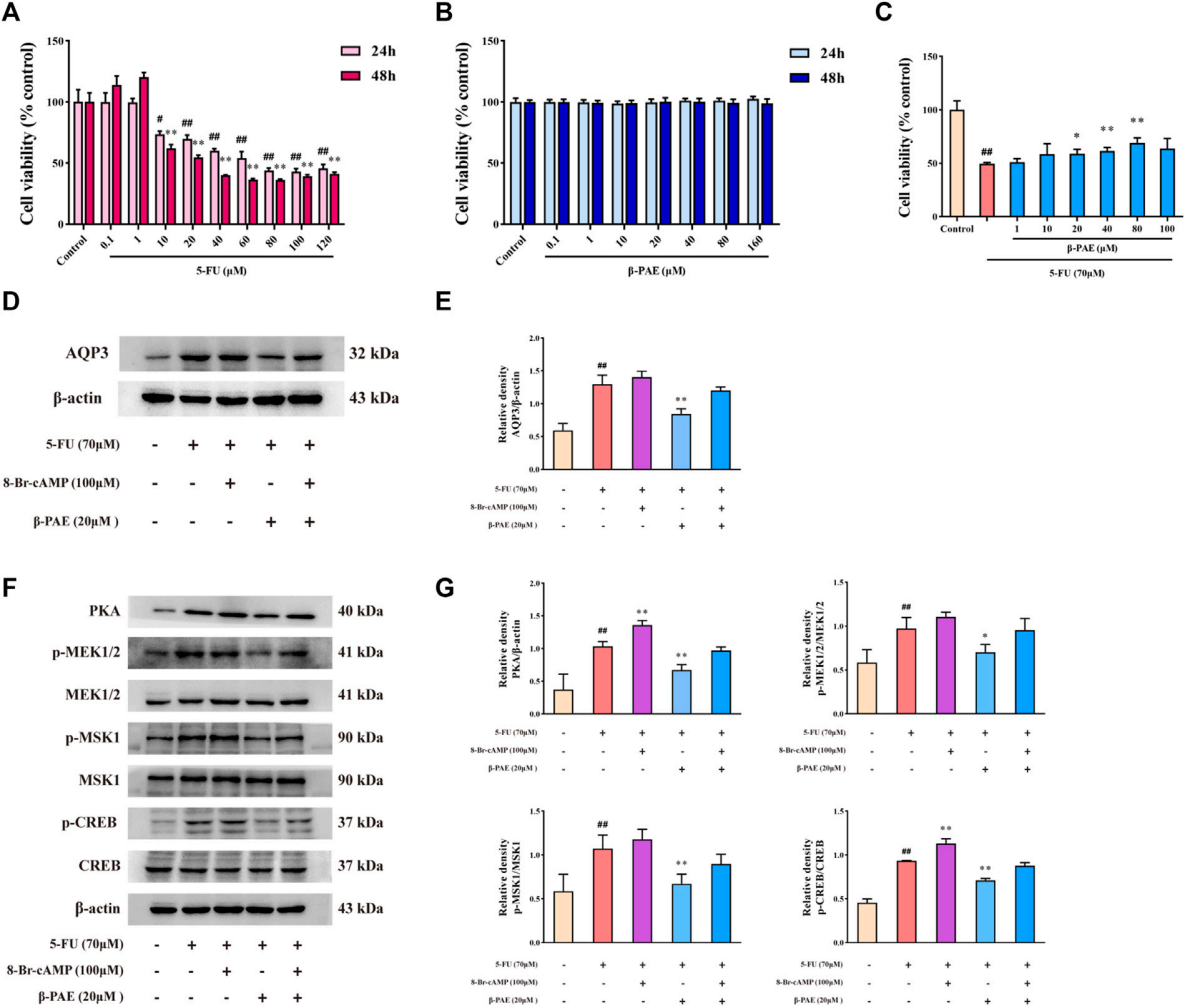
Effects of *β*-PAE on AQP3 expression and PKA/CREB signaling pathway in IEC-6 cells. Cell viability following treatment with different doses of **(A)** 5-FU for 24 and 48 h and **(B)** *β*-PAE for 24 and 48 h; **(C)** *β*-PAE enhanced 5-FU-injured cell viability; **(D,E)** AQP3 expression; **(F,G)** PKA, p-MEK1/2, MEK1/2, p-MSK1, MSK1, p-CREB, and CREB expression. Data are shown as mean ± SD (*n* = 3). ^##^ *p* < 0.01 versus control group; ^*^ *p* < 0.05, ^**^ *p* < 0.01 versus 5-FU group.

Treatment with 10 μM or higher concentration of 5-FU significantly decreased IEC-6 cell viability over 24 and 48 h, and the IC50 was 70 μM at 24 h (Figure 2A). The treatment concentration of *β*-PAE selected showed no cytotoxicity in IEC-6 cells in 24 and 48 h (Figure 2B). Therefore, 70 μM 5-FU was used in the subsequent steps. Low IEC-6 cell viability caused by treatment with 70 μM 5-FU was restored by exposure to *β*-PAE. Herein, 20, 40, and 80 μM *β*-PAE significantly improved cell viability to 58.93, 61.43, and 69.05%, respectively (Figure 2C, *p* < 0.05). These results suggested the doses of 5-FU and *β*-PAE be used in the *in vitro* studies.

Compared with the control group, 5-FU notably enhanced AQP3 and PKA expression, increased the ratio of p-MEK1/2/MEK1/2, p-MSK1/MSK1, and p-CREB/CREB (Figures 2D–G, *p* < 0.01). The PKA agonist 8-Br-cAMP was able to amplify these trends (Figures 2D–G). As expected, *β*-PAE strikingly suppressed AQP3 and PKA expression, inhibited MEK1/2, MSK1, and CREB phosphorylation when compared with the 5-FU group (Figures 2D–G, *p* < 0.01). Nevertheless, 8-Br-cAMP counteracted the effects of *β*-PAE *via* upregulating the expression or phosphorylation of the related proteins.

### *β*-Patchoulene Improved Body Characteristics and Small Intestine Histopathology in Intestinal Mucositis-Induced Rats

**FIGURE 3:**
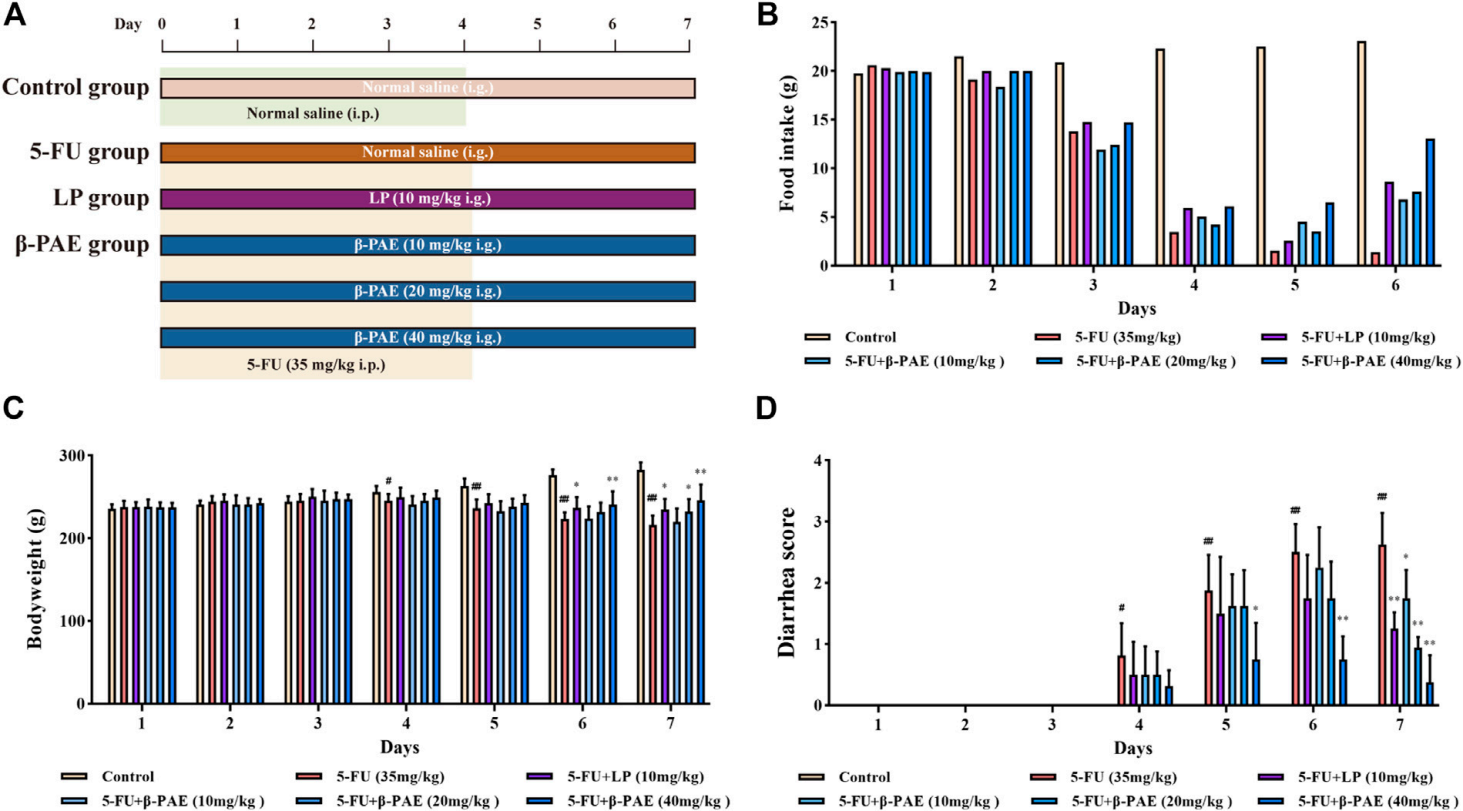
Effects of *β*-PAE on the body characteristics. **(A)** Study scheme of *β*-PAE treatment in IM rats; **(B)** Food intake; **(C)** Bodyweight; **(D)** Diarrhea scores. Data are shown as mean ± SD (*n* = 8). ^#^ *p* < 0.05, ^##^ *p* < 0.01 versus control group; ^*^ *p* < 0.05, ^**^ *p* < 0.01 versus 5-FU group.

Then the study about the effect of *β*-PAE in IM-induced rats was carried out (Figure 3A). Food consumption on Days 1 and 2 was minimally changed between the groups, which indicated there was no significant change in the IM-induced rat body weight from Days 1–3. The food intake between the control group and other groups began to differentiate on the following days (Figure 3B), and similarly bodyweight and diarrhea scores (Figures 3D,C). The maximum side effects of 5-FU occurred on Day 7 with the lowest body weight, food intake, and the worst diarrhea symptoms in IM-induced rats without treatment. In contrast, IM-induced rats orally treated with *β*-PAE or LP resisted the adverse effects of 5-FU and showed improved body weight, food intake, and diarrhea symptoms. On Day 7, the bodyweight of the LP group and *β*-PAE (20, 40 mg/kg) groups were significantly increased (Figure 3C, all *p* < 0.05) with diet recovery compared with the 5-FU group, respectively. Besides, all treatment groups showed a marked improvement of diarrhea symptoms compared with the 5-FU group (Figure 3D, *p* < 0.05)

**FIGURE 4:**
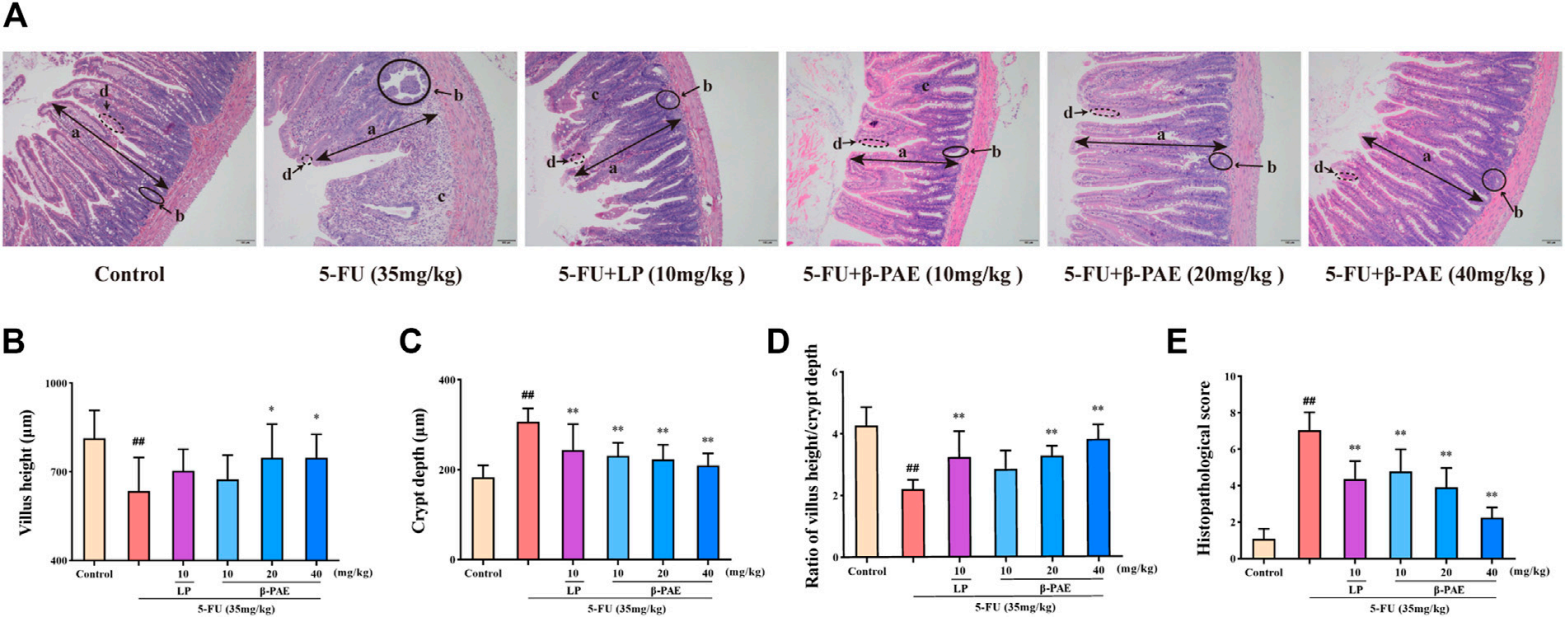
Effects of *β*-PAE on small intestinal histopathology. **(A)** Small intestine sections showed by H&E staining (magnification 100x), the symbols are as follows: (a) villus; (b) crypt lumen; (c) infiltration area; (d) microvillus; **(B)** Villus height; **(C)** Crypt depth; **(D)** Ratio of villus height/crypt depth; **(E)** The histopathological score of small intestine H&E staining. Data are shown as mean ± SD (*n* = 6). ^#^ *p* < 0.05, ^##^ *p* < 0.01 versus control group; ^*^ *p* < 0.05, ^**^ *p* < 0.01 versus 5-FU group.

As for histopathology, compared with control group, the structure of the small intestine was seriously damaged after 5-FU administration (Figure 4A), as evidence by the very short villus heights (Figure 4B, *p* < 0.01), swollen crypt lumens (Figure 4C, *p* < 0.01), low ratio of villus height/crypt lumen (Figure 4D, *p* < 0.01), and large infiltration areas. Therefore, these were graded the worst score in the histopathological assay (Figure 4E, *p* < 0.01). By comparison, the LP group and the different doses of *β*-PAE treatment exhibited a better histological appearance of the small intestine. They showed normal villus, small crypt, well-ordered glands, and indistinct infiltration areas. Hence, in terms of histopathological scores, the LP and *β*-PAE groups were markedly decreased compared with the5 -FU group (Figure 4E, all *p* < 0.01).

### *β*-Patchoulene Inhibited Aquaporin3 Expression *via* Inactivating the cAMP/Protein Kinase A/cAMP-Response Element-Binding Protein Signaling Pathway in Intestinal Mucositis-Induced Rats

**FIGURE 5:**
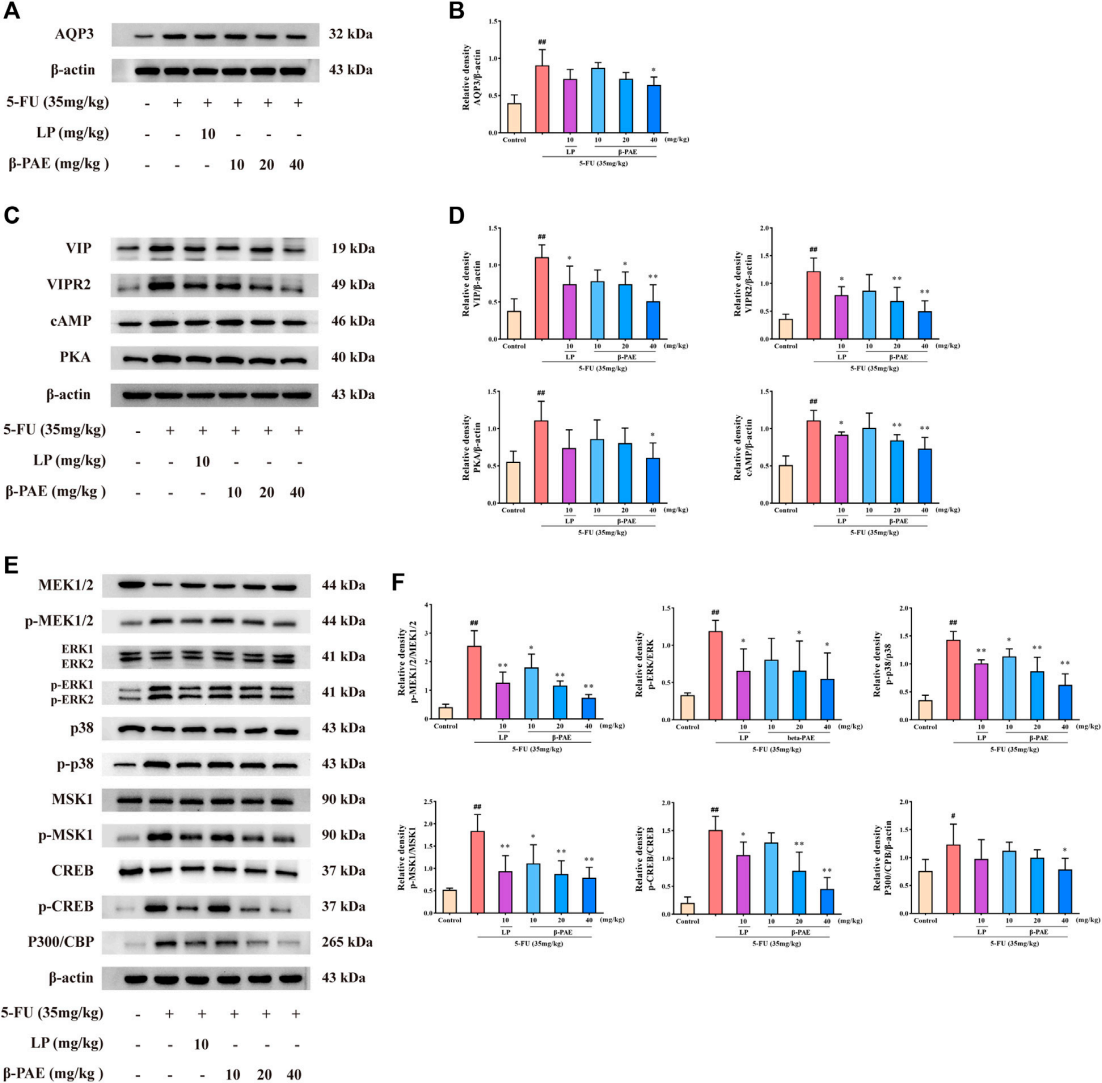
Effects of *β*-PAE on AQP3 expression and cAMP/PKA/CREB signaling pathway-related proteins. **(A,B)** AQP3 expression; **(C,D)** VIP, VIPR2, cAMP and PKA expression; **(E,F)** MEK1/2, p-MEK1/2, ERK, p-ERK, p-p38, p38, MSK1, p-MSK1, CREB, p-CREB, and P300/CBP expression. Data are shown as mean ± SD (*n* = 3). ^#^ *p* < 0.05, ^##^ *p* < 0.01 versus control group; ^*^ *p* < 0.05, ^**^ *p* < 0.01 versus 5-FU group.

Compared with the control group, AQP3, VIP, VIPR2, cAMP, and PKA expression were markedly increased in the 5-FU group (Figures 5A–D, *p* < 0.01). Furthermore, MEK1/2, ERK1/2, p38, MSK1, and CREB proteins were significantly phosphorylated, and P300/CBP expression was enhanced in the 5-FU group (Figures 5E,F, *p* < 0.05). Compared with the 5-FU group, *β*-PAE treatment inhibited the overexpression of all these proteins. Among the three doses of *β*-PAE tested, 40 mg/kg of *β*-PAE achieved a better inhibition of protein phosphorylation or expression. Especially, it significantly (*p* < 0.05) decreased AQP3, PKA, and P300/CBP expression while other doses of *β*-PAE and LP could not.

### *β*-Patchoulene Suppressed Pro-inflammatory Cytokines Levels

**FIGURE 6:**
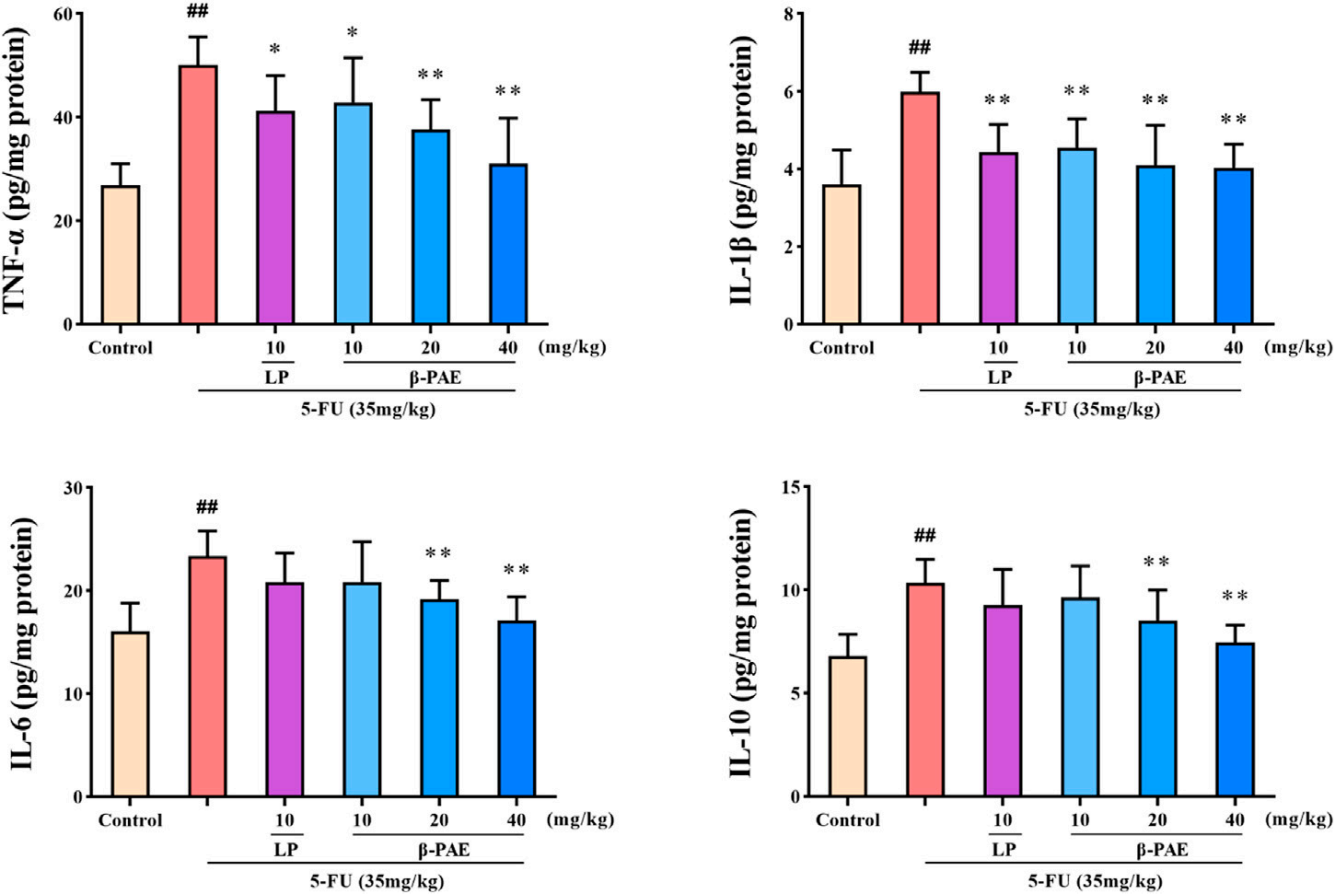
Effects of *β*-PAE on intestinal TNF-α, IL-1β, IL-6 and IL-10 expression. Data are shown as mean ± SD (*n* = 8). ^##^ *p* < 0.01 versus control group; ^*^ *p* < 0.05, ^**^ *p* < 0.01 versus 5-FU group.

TNF-α, IL-1β, IL-6, and IL-10 were significantly increased to 50.11, 5.99, 23.38, and 10.33 pg/mg protein after 5-FU administration, respectively (Figure 6, *p* < 0.01). However, *β*-PAE (20 and 40 mg/kg) significantly decreased the abnormal cytokine levels (*p* < 0.01) induced by 5-FU. In particular, 40 mg/kg *β*-PAE downregulated the levels of TNF-α, IL-1β, IL-6, and IL-10 to 31.02, 4.04, 17.10, and 7.46 pg/mg protein, respectively.

### *β*-Patchoulene Improved the Expression of Mucin-2 Mucin and Goblet Cell Injury

**FIGURE 7:**
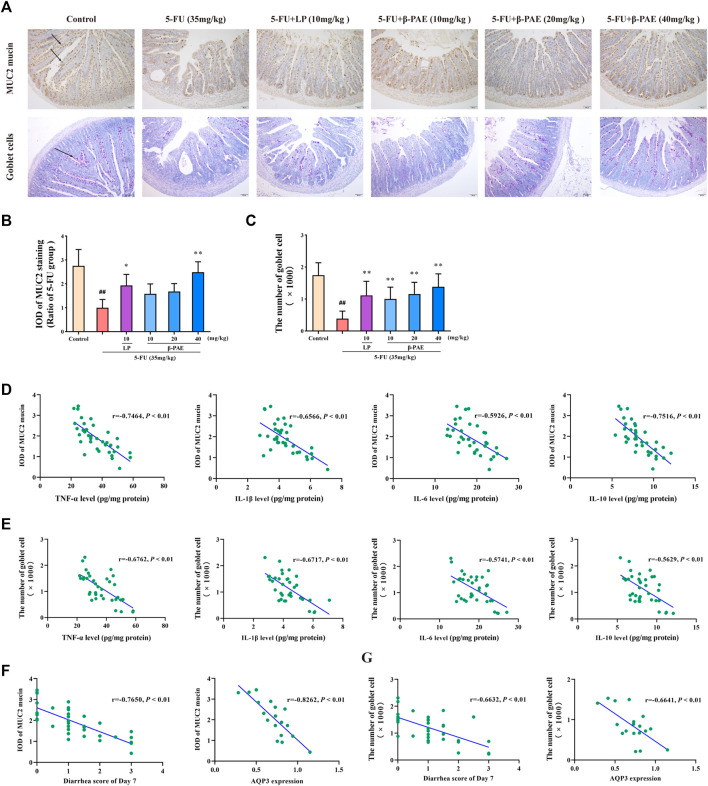
Effects of β-PAE on mucus layer. **(A)** MUC2 mucin protein and goblet cells in the small intestine (magnification 100x), the arrows indicate positive protein expression; **(B)** IOD of MUC2 mucin expression; **(C)** The number of goblet cells; Spearman’s correlation analysis **(D)** between MUC2 mucin protein expression and inflammatory cytokines level; **(E)** between goblet cells and inflammatory cytokines level; **(F)** between MUC2 mucin expression and diarrhea symptoms; and AQP3 levels; **(G)** between goblet cells and diarrhea symptoms; and AQP3 levels. Data are shown as mean ± SD (*n* = 6). ^##^ *p* < 0.01 versus control group; ^*^ *p* < 0.05, ^**^ *p* < 0.01 versus 5-FU group.

Less MUC2 mucin-positive expression and goblet cells-positive expression was detected in the 5-FU group (Figure 7A). The IOD of MUC2 mucin and the number of goblet cells in the 5-FU-treated group were significantly lower than those in the control group (Figures 7B,C, *p* < 0.01). *β*-PAE and LP were able to recover MUC2 mucin expression and protect goblet cells. In particular, compared with the 5-FU-treated group, 40 mg/kg *β*-PAE significantly promoted MUC2 mucin expression and the number of goblet cells to 2.49 and 1,382, respectively (Figures 7B,C, *p* < 0.01).

Correlation analysis further revealed that the abovementioned inflammatory cytokines negatively regulated the expression of MUC2 mucin and goblet cells (Figures 7D,E). Besides, MUC2 mucin (Figure 7F) remarkably correlated with diarrhea symptoms (|*r*| > 0.7) and AQP3 expression (|*r*| > 0.8), and living goblet cells (Figure 7G) also showed a great correlation with diarrhea symptoms (|*r*| > 0.6) and AQP3 levels (|*r*| > 0.6).

### *β*-Patchoulene Showed Better Outcomes Than PA on the Regulation of Water Transport by Suppressing Aquaporin3 Expression in Intestinal Mucositis-Induced Rats

**FIGURE 8:**
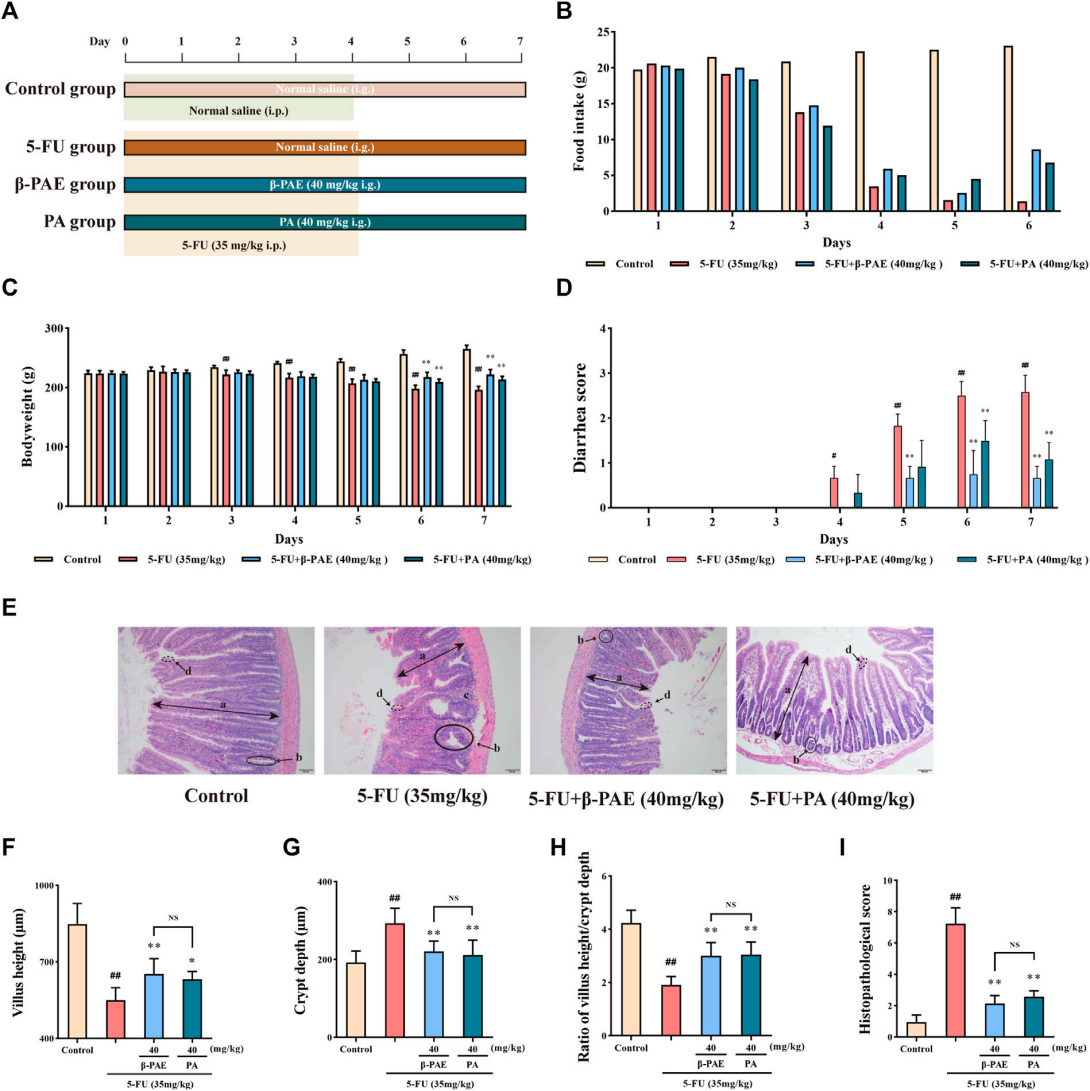
Effects of *β*-PAE and PA on the body characteristics and small intestine histopathology. **(A)** Comparative study scheme of *β*-PAE and PA treatment in IM rats; **(B)** Food intake; **(C)** Bodyweight; **(D)** Diarrhea scores; **(E)** Small intestine sections showed by H&E staining (magnification 100x), the symbol are as follows: (a) villus; (b) crypt lumen; (c) infiltration area; (d) microvillus; **(F)** Villus height; **(G)** Crypt depth; **(H)** Ratio of villus height/crypt depth; **(I)** The histopathological score of small intestine H&E staining. Data are shown as mean ± SD (*n* = 6−7). ^#^ *p* < 0.05, ^##^ *p* < 0.01 versus control group; ^*^ *p* < 0.05, ^**^ *p* < 0.01 versus 5-FU group. NS, not statistically significant.

A comparative study between *β*-PAE and PA was carried out in IM-induced rats (Figure 8A). *β*-PAE and PA both increased food intake (Figure 8B), body weight (Figure 8C, *p* < 0.01), and ameliorated diarrhea (Figure 8D, *p* < 0.01) in IM-induced rats compared with the 5-FU group on Days 6 and 7. *β*-PAE and PA-treated groups showed a similar integrated epithelial structure (Figure 8E), whereas there was no significant difference in the quantification of villus height and crypt depth (Figures 8F–H). As a result, both obtained similar scores in the histopathological analysis (Figure 8I).

**FIGURE 9:**
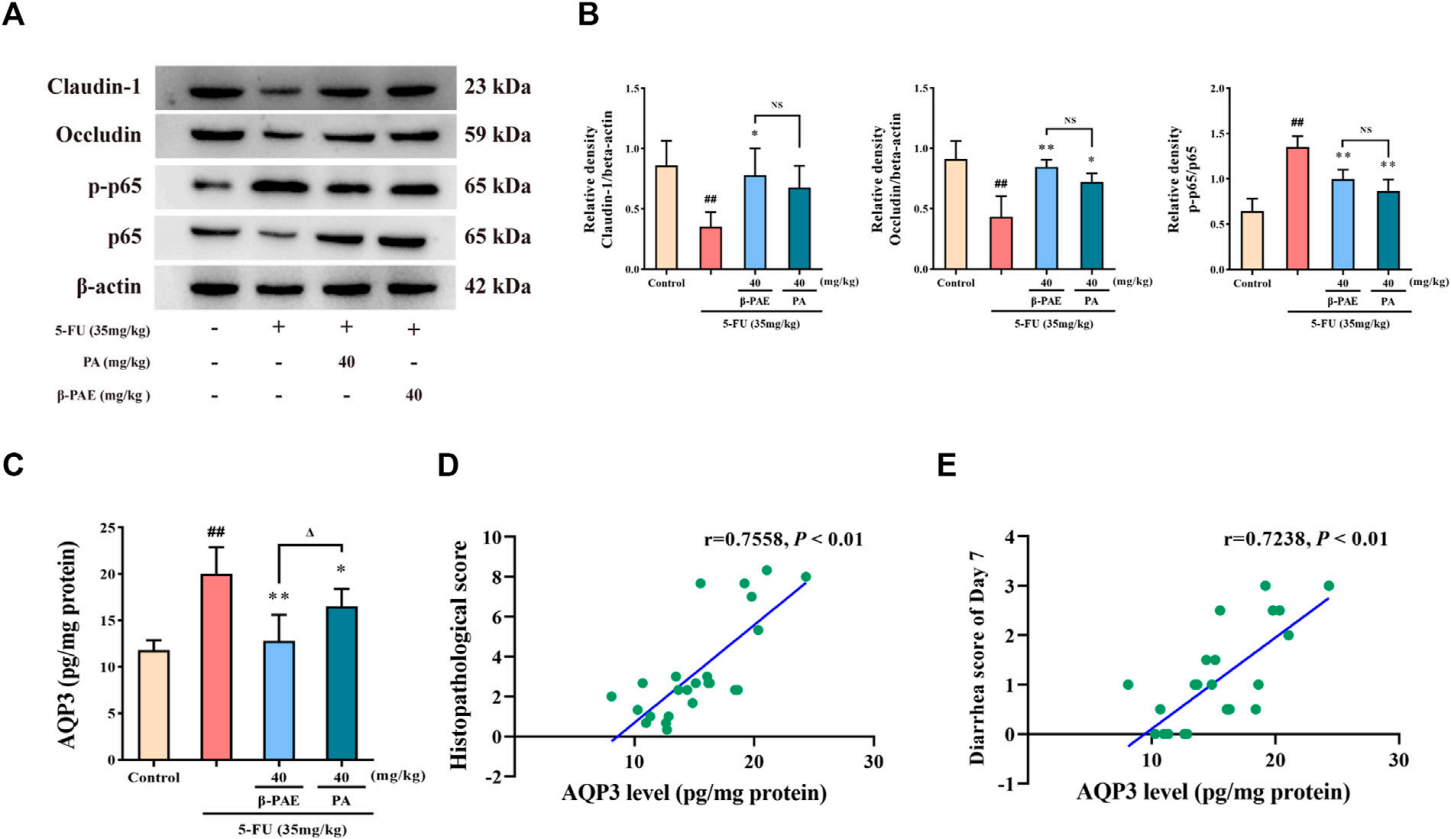
Comparative study of the effects of *β*-PAE and PA on the epithelial barrier, inflammatory response, and water transport. **(A,B)** Claudin-1, occludin, p-p65 and p65 expression; **(C)** AQP3 expression; **(D)** Spearman correlation analysis between AQP3 level and histopathological score; **(E)** Spearman correlation analysis between AQP3 level and diarrhea scores on Day 7. Data are shown as mean ± SD (*n* = 3–6). ^##^ *p* < 0.01 versus control group; ^*^ *p* < 0.05, ^**^ *p* < 0.01 versus 5-FU group; ^Δ^ *p* < 0.05 versus PA group. NS: not statistically significant.

Pathologically, *β*-PAE and PA showed a similar effect on maintaining the integrity of the intestinal epithelial barrier and improving inflammation. Compared with the 5-FU-treatment group, *β*-PAE and PA treatment both significantly enhanced occludin expression (Figures 9A,B, *p* < 0.05) and reduced the ratio of p-p65/p65 (Figures 9A,B, *p* < 0.01). However, there was a significant gap in the inhibition of AQP3 expression (Figure 9C). *β*-PAE and PA both showed significant inhibitory effects when compared with the 5-FU group (*p* < 0.05), but *β*-PAE showed a better effect than PA on reducing AQP3 expression (*p* < 0.05). This indicated that *β*-PAE focused more on regulating water transport than PA. The correlation analysis also showed AQP3 levels were strongly related to the histopathological score and diarrhea scores (Figures 9D,E, |*r*| > 0.7).

## Discussion

This study revealed that *β*-PAE effectively improved body weight, food intake, and diarrhea symptoms in 5-FU-induced IM rats. Pathologically, improving water transport, inflammation, and mucus barrier injury was pivotal for processes involved in *β*-PAE amelioration of diarrhea and the cAMP/PKA/CREB signaling pathway played an important role in this activity.

Diarrhea is a common symptom in IM patients and is associated with water transport dysfunction (Camilleri et al., 2012; Ikarashi et al., 2016). AQP is a transmembrane water channel protein and is critical for regulating water fluid homeostasis (Laforenza, 2012). High expression of AQPs has been identified as a co-factor in the etiopathogenesis of some gastroenteric disorders (Guttman et al., 2007). Recently, AQP3 has become a potential therapeutic target due to its pivotal role in water and glycerol transport in the presence of diarrhea (Liu et al., 2014; Ikarashi et al., 2016; Kon et al., 2018), and its absence facilitates water and glycerol transport dysfunction in AQP3 knock-out mice (Levin and Verkman, 2006). 5-FU significantly enhanced AQP3 expression, but *β*-PAE reversed it in cells. A similar result reappeared in IM-induced rats. Interestingly, 5-FU-induced IM in rats produced severe diarrhea, while *β*-PAE effectively alleviated diarrhea in this model. These results indicated that 5-FU impaired water transport by increasing AQP3 expression and thus induced severe diarrhea. *β*-PAE treatment decreased AQP3 abnormal expression to restore water transport and relieved diarrhea symptoms.

The cAMP/PKA/CREB signaling pathway participates in AQPs expression (Wang and Zheng, 2011; Zhang et al., 2020; Kim et al., 2021). cAMP is extensively expressed in cells and directly regulates various biological processes or behaviors of cells, including cell metabolism, ion channel activation, gene expression, cell growth, differentiation, and apoptosis (Chin et al., 2002). PKA is a major target of cAMP. When the level of cAMP increases in cells, it binds PKA substrates and then phosphorylates them (Kandel, 2012). The CREB family proteins are well-characterized PKA substrates, and CREB phosphorylation mediates P300/CBP and initiates gene transcription, thereby regulating cell differentiation, proliferation, apoptosis, and metabolism (Siu and Jin, 2007; Lee et al., 2010; Xiao et al., 2010). In addition to the major PKA kinase, other kinases such as MEK, MSK also directly phosphorylate CREB (Wiggin et al., 2002; Li et al., 2017). The cAMP/PKA/CREB signaling pathway is activated by increasing cAMP, PKA, p-MEK1/2, p-MSK1, and p-CREB expression in cells or rats after 5-FU treatment. *β*-PAE treatment decreased the expression of the abovementioned proteins or their phosphorylation to inactivate the cAMP/PKA/CREB signaling pathway and thus downregulated AQP3 expression.

The vasoactive intestinal polypeptide (VIP) is an abundant gut peptide hormone. It controls neuronal, epithelial, and endocrine cell functions, which in turn regulate ion secretion, nutrient absorption, and gut motility (Iwasaki et al., 2019). VIP activates its receptors VIPR2 to stimulate intestinal gland secretion activity and affects intestinal mucus content to induce diarrhea (Fung et al., 2014). Specifically, VIP induces MEK/ERK and p38 MAPK phosphorylation to mediate MSK1 and CREB expression and MUC2 gene transcription (Fernandez et al., 2005; Hokari et al., 2005). Indeed, VIP, VIPR2, p-ERK, and p-p38 MAPK expression were upregulated in IM-induced rats, but *β*-PAE treatment effectively reduced the overexpression of these proteins, and thus down-regulated p-MSK1 and CREB phosphorylation.

Intestinal barrier damage and inflammatory response dysfunction were also observed in IM development (Camilleri et al., 2012; Wu et al., 2020). Tight junctions (TJs) play an important role in maintaining epithelial permeability that simultaneously occludes the paracellular space, dictates ion flux across the tissue, and maintains cellular polarity (Odenwald and Turner, 2017). TJs leakage disrupts gastrointestinal homeostasis and makes exogenous toxins flow into circulation, then induces the inflammatory response (Groschwitz and Hogan, 2009). VIP treatment activates ERK, p38 MAPK, and NF-κB signaling pathways, and these factors facilitate the release of downstream pro-inflammatory cytokines (Hokari et al., 2005; Hoesel and Schmid, 2013; Dong et al., 2020). Besides, NF-κB p65 also induces CBP/P300 expression to regulate the transcription of pro-inflammatory mediators (Wen et al., 2010). *β*-PAE activity in UC mice recovered TJs by increasing the expression of claudin-1, occludin proteins. Meanwhile, by decreasing VIP expression, *β*-PAE inhibited the phosphorylation of ERK, p38 MAPK, and NF-κB p65 and blocked the release of pro-inflammatory cytokines.

Inflammation then disrupts intestinal homeostasis, exacerbates MUC2 misfolding, and thus results in mucus layer depletion (Sanchez de Medina et al., 2014). The well-functioning mucus layer is capable of retaining water better and reduced water transport, while its depletion weakens water-holding capacity and disturbs water transport (Pelaseyed et al., 2014). Because of the dysfunction in gastrointestinal water transport, severe pathological diarrhea events are observed in IM patients. Unsurprisingly, with increasing pro-inflammatory levels, lower expression of MUC2 mucin and fewer goblet cells were identified in IM-induced rats. Conversely, because of *β*-PAE’s marvelous anti-inflammatory properties, it significantly enhanced MUC2 mucin expression and goblet cells viability in the intestine. The correlation analysis revealed a negative relationship between MUC2 mucin, goblet cells, and inflammatory cytokines levels.

The integrated mucus layer is a barrier to prevent water loss and to remove inhaled foreign substances such as microbes, inflammatory cells, and pollutant particles (Ma et al., 2018). When IM occurs, mucus layer depletion with diminished expression of MUC2 mucins and goblet cells viability occurs. Dysfunction of the mucus layer, in turn, worsens inflammation and weakens the water-holding capacity of the gastrointestinal tract, induces AQP3 overexpression, and finally leads to severe diarrhea in IM-induced rats. As mentioned above, *β*-PAE attenuated the inflammatory response, improved the mucus layer function, reduced AQP3 expression, and thus restored the rat’s water-holding capacity. The correlation analysis also indicated AQP3 and diarrhea symptoms were closely related to mucus layer function. Altogether, the strong correlation between inflammatory cytokines and mucus layer function mentioned in the previous paragraph and the improved intestinal inflammation and mucus layer function may represent a bypass mechanism for *β*-PAE to regulate AQP3 expression.

Furthermore, we also determined that *β*-PAE exhibited superior effects than PA on water transport by inhibiting AQP3 overexpression. As for intestinal barrier and inflammation, both improved claudin-1 and occludin expression, as well as NF-κB p65 phosphorylation, but no significant differences were observed. Accordingly, the significant effect of patchouli oil on mediating water transport function was mainly driven by *β*-PAE, and secondly by PA.

In conclusion, this study revealed a marked correlation between AQP3 and diarrhea, AQP3 and histopathological score, respectively. These data provided strong evidence supporting the importance of AQP3 in diarrhea throughout IM development. Overexpression and ectopic expression of AQP3 have been observed in several cancers, where it has been shown to induce metastasis, proliferation, and epithelial-to-mesenchymal transition (EMT) (Chen et al., 2014; Huang et al., 2015; Marlar et al., 2017). These properties strongly indicated that AQP3 functions not only as a biomarker in several cancers but is also a potential gatekeeper in IM. Future study is required to confirm these findings.

## Data Availability

The datasets presented in this study can be found in online repositories. The names of the repository/repositories and accession number(s) can be found in the article/Supplementary Material.

## Abbreviations

5-FU, 5-fluorouracil; AQP, aquaporin; β-PAE, β-patchoulene; BSA, bovine serum albumin; cAMP, cyclic adenosine monophosphate; CREB, cAMP-response element-binding protein; ERK, extracellular regulated protein kinases; GIT, gastrointestinal tract; H&E, hematoxylin, and eosin; IBD, inflammatory bowel disease; IEC, intestinal epithelial cell; IC50, 50% inhibitory concentration; IHC, immunohistochemistry; IL-1β, interleukin-1 β; IL-6, interleukin-6; IL-10, interleukin-10; IM, intestinal mucositis; IOD, integrated optical density; LP, Loperamide hydrochloride; MUC2, mucin-2; PA, patchouli alcohol; PAS, Periodic Acid-Schiff; PKA, protein kinase A; qRT-PCR, quantitative real-time PCR; TCM, traditional Chinese medicine; TJs, tight junctions; TNF-α, tumor necrosis factor-alpha; VIP, vasoactive intestinal polypeptide
