# Supplementary figures and images for: β-Patchoulene Ameliorates Water Transport and the Mucus Barrier in 5-Fluorouracil-Induced Intestinal Mucositis Rats via the cAMP/PKA/CREB Signaling Pathway
Source: Front Pharmacol. 2021 Aug 25;12:689491. doi: 10.3389/fphar.2021.689491 (PMC8424048; doi:10.3389/fphar.2021.689491)

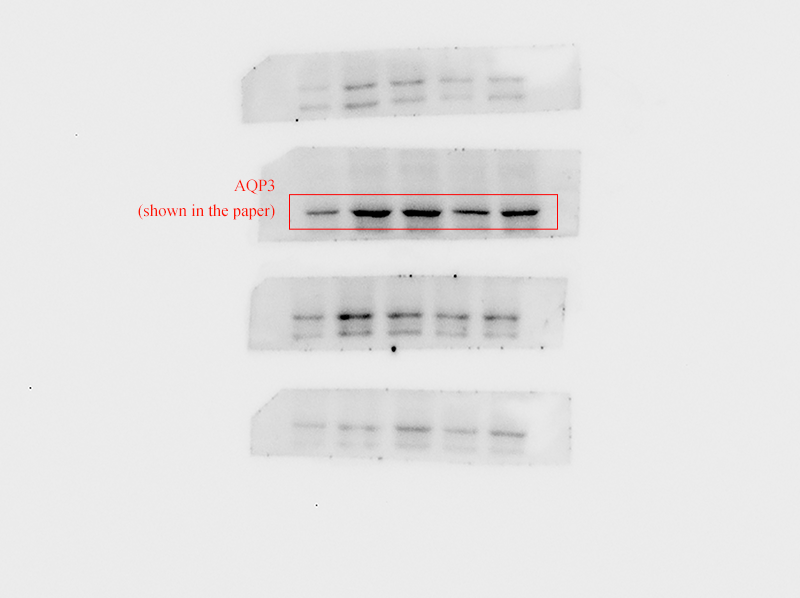

Supplement: Supplementary file 1 [file DataSheet1.ZIP › Original western blot-cells/AQP3-A.tif]

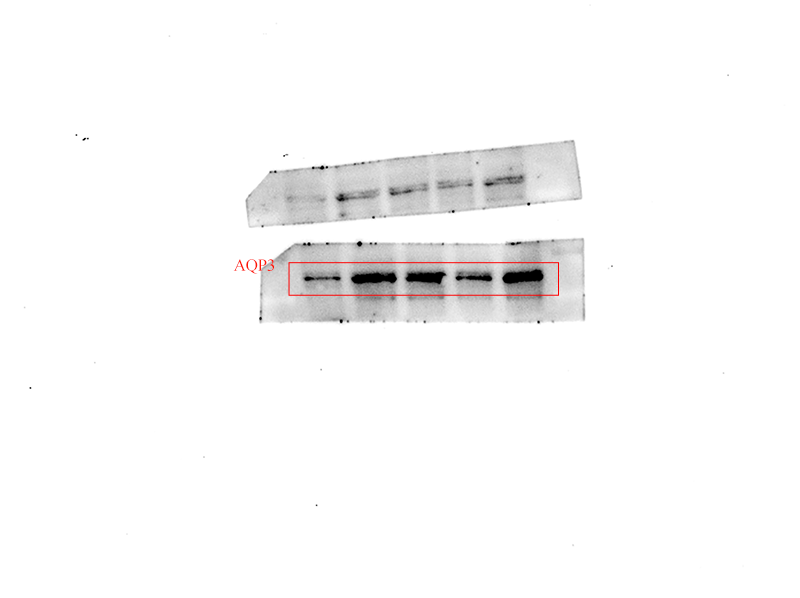

Supplement: Supplementary file 1 [file DataSheet1.ZIP › Original western blot-cells/AQP3-B.tif]

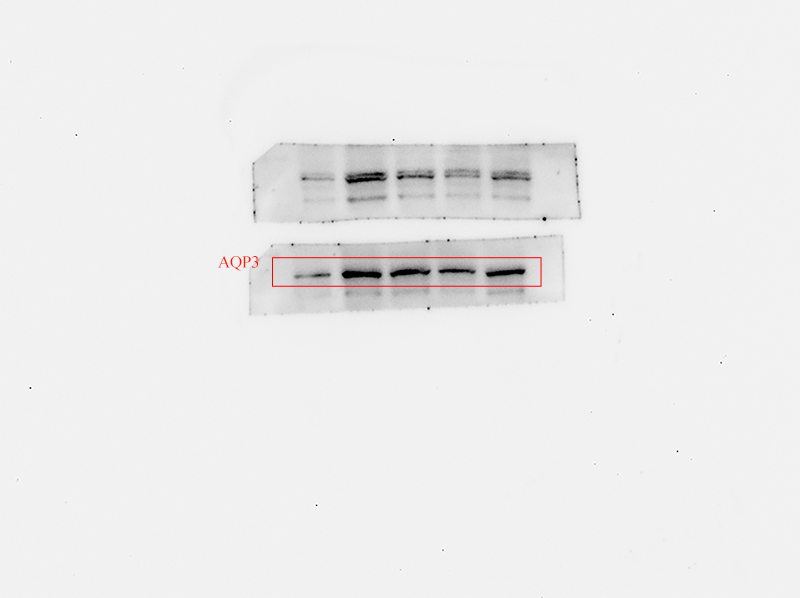

Supplement: Supplementary file 1 [file DataSheet1.ZIP › Original western blot-cells/AQP3-C.tif]

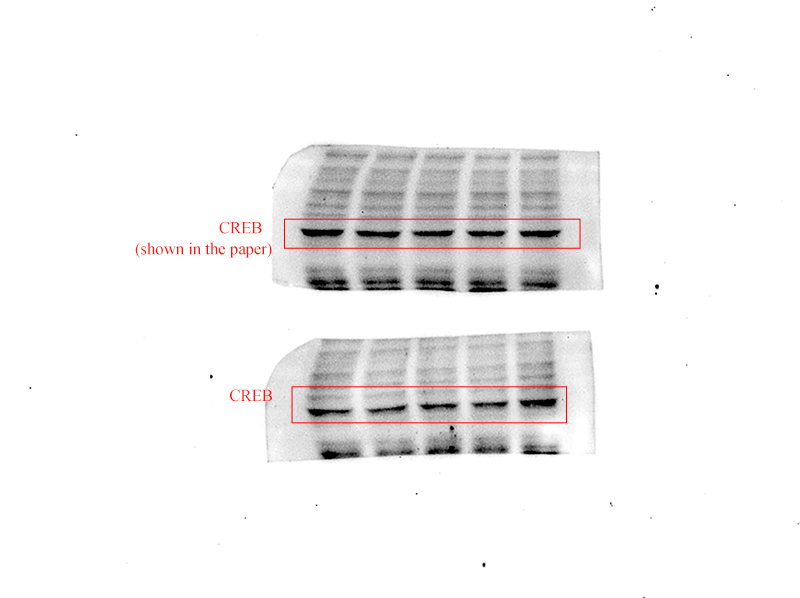

Supplement: Supplementary file 1 [file DataSheet1.ZIP › Original western blot-cells/CREB-A.tif]

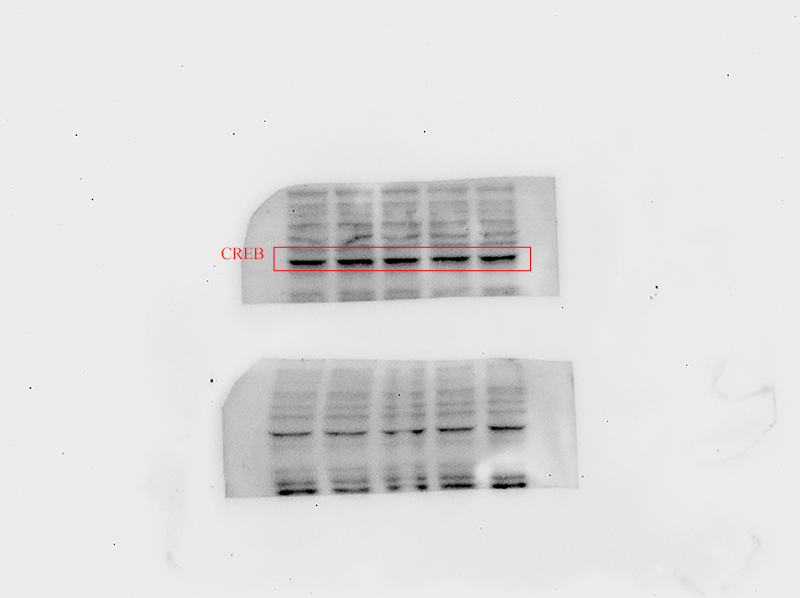

Supplement: Supplementary file 1 [file DataSheet1.ZIP › Original western blot-cells/CREB-B.tif]

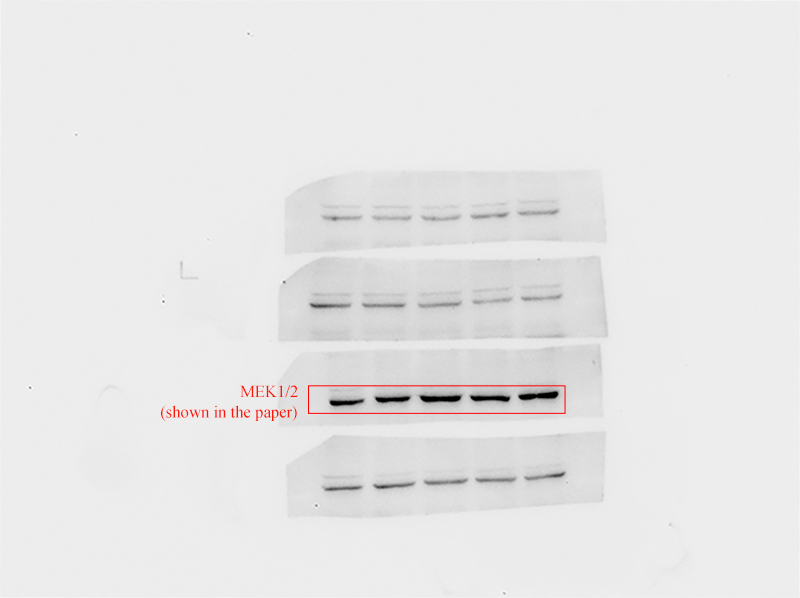

Supplement: Supplementary file 1 [file DataSheet1.ZIP › Original western blot-cells/MEK-A.tif]

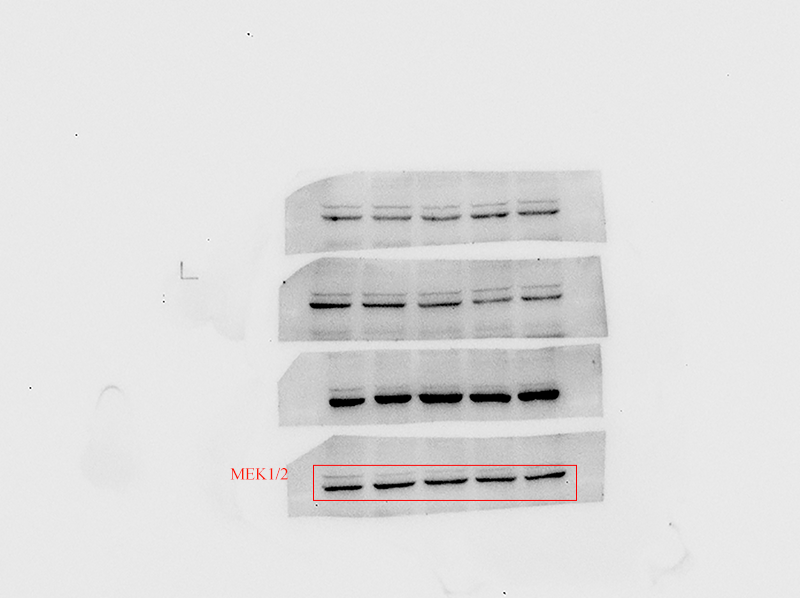

Supplement: Supplementary file 1 [file DataSheet1.ZIP › Original western blot-cells/MEK-B.tif]

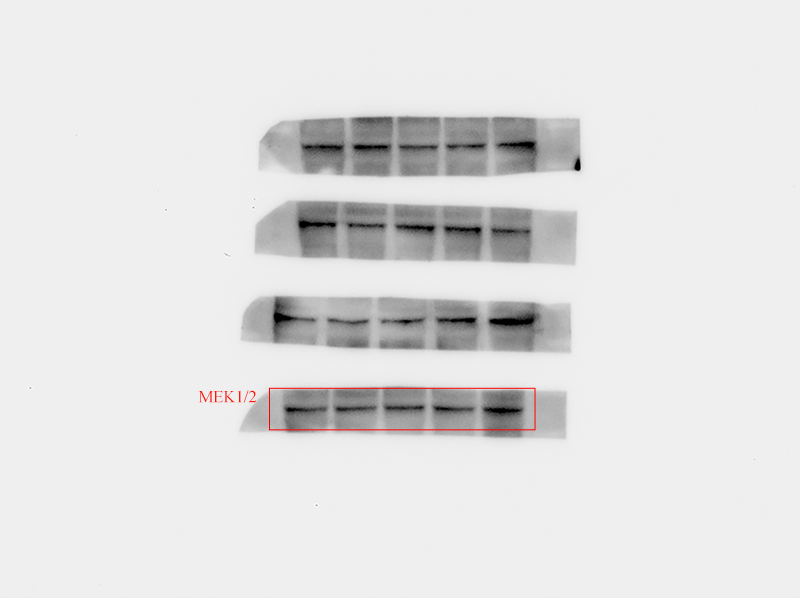

Supplement: Supplementary file 1 [file DataSheet1.ZIP › Original western blot-cells/MEK-C.tif]

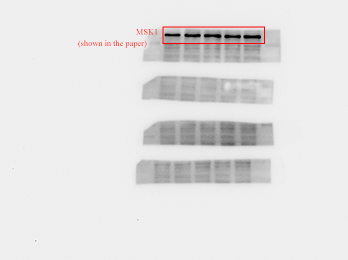

Supplement: Supplementary file 1 [file DataSheet1.ZIP › Original western blot-cells/MSK-A.tif]

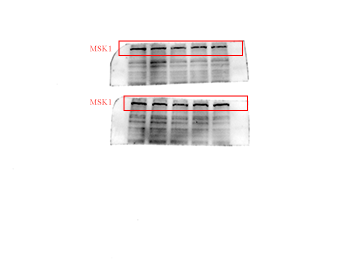

Supplement: Supplementary file 1 [file DataSheet1.ZIP › Original western blot-cells/MSK-B.tif]

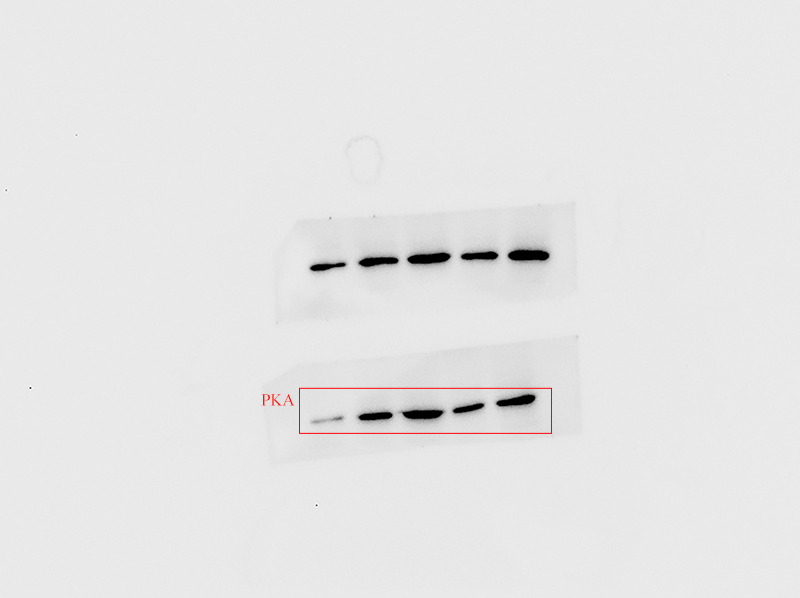

Supplement: Supplementary file 1 [file DataSheet1.ZIP › Original western blot-cells/PKA-A.tif]

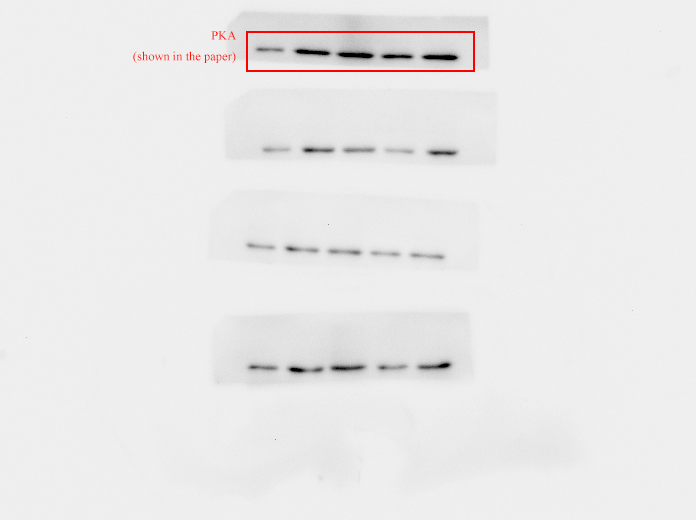

Supplement: Supplementary file 1 [file DataSheet1.ZIP › Original western blot-cells/PKA-B.tif]

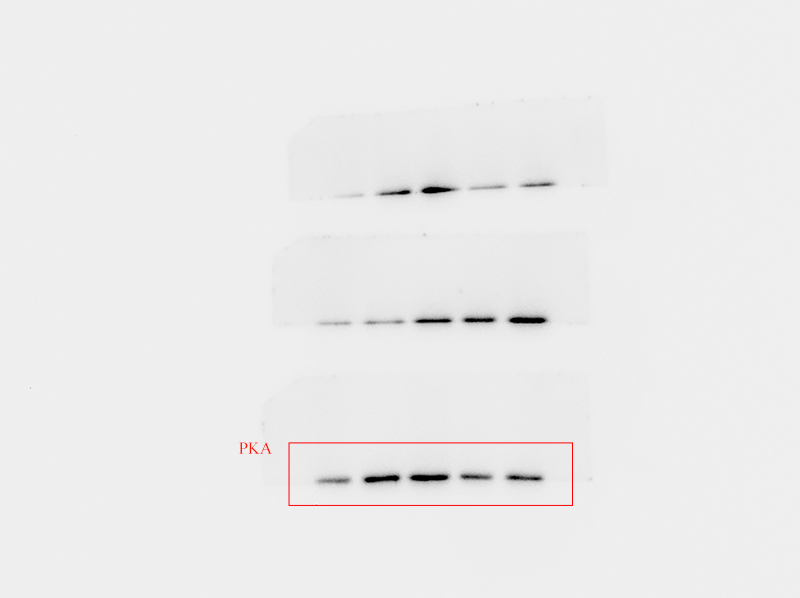

Supplement: Supplementary file 1 [file DataSheet1.ZIP › Original western blot-cells/PKA-C.tif]

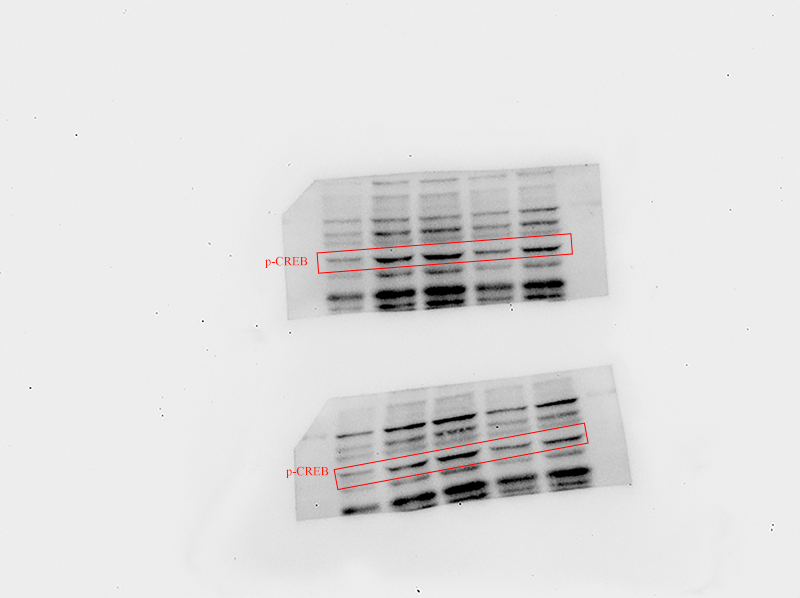

Supplement: Supplementary file 1 [file DataSheet1.ZIP › Original western blot-cells/p-CREB-A.tif]

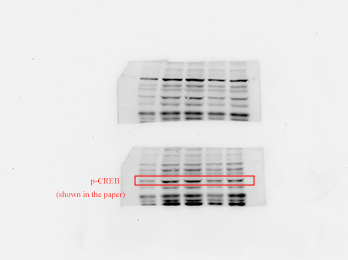

Supplement: Supplementary file 1 [file DataSheet1.ZIP › Original western blot-cells/p-CREB-B.tif]

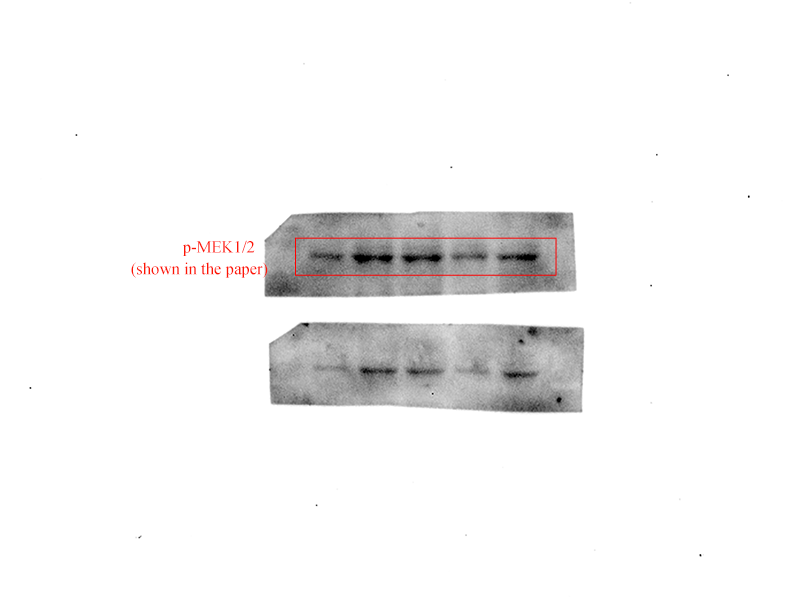

Supplement: Supplementary file 1 [file DataSheet1.ZIP › Original western blot-cells/p-MEK-A.tif]

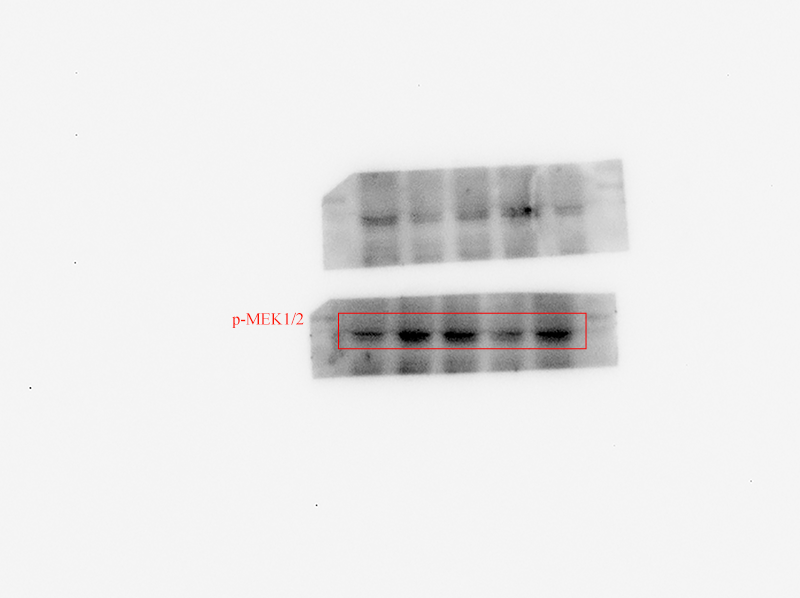

Supplement: Supplementary file 1 [file DataSheet1.ZIP › Original western blot-cells/p-MEK-B.tif]

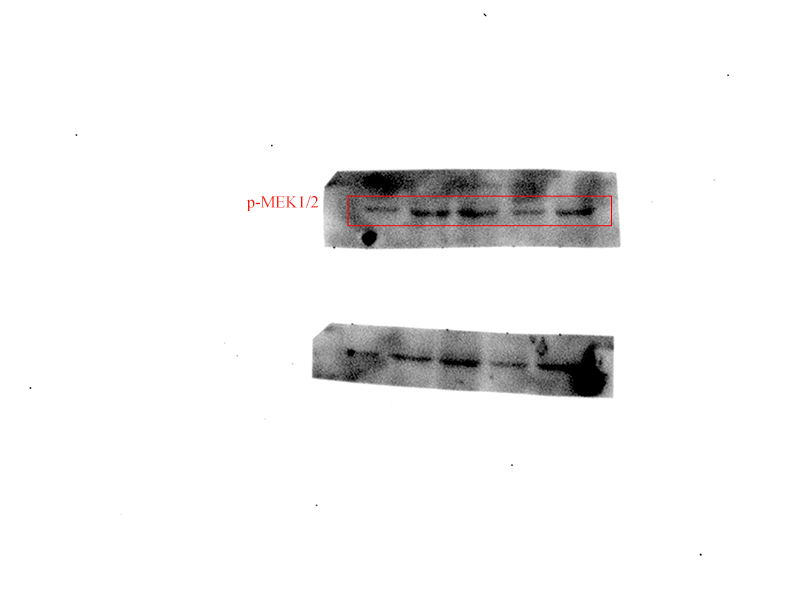

Supplement: Supplementary file 1 [file DataSheet1.ZIP › Original western blot-cells/p-MEK-C.tif]

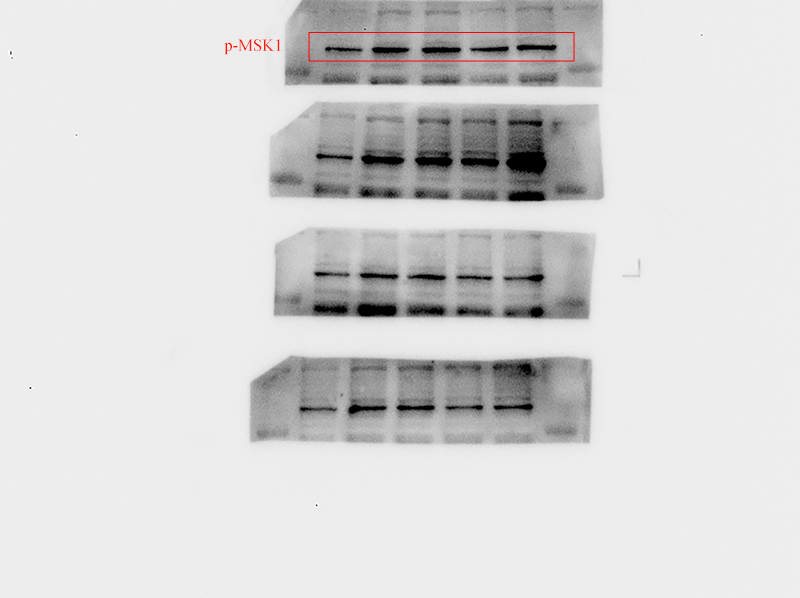

Supplement: Supplementary file 1 [file DataSheet1.ZIP › Original western blot-cells/p-MSK-A.tif]

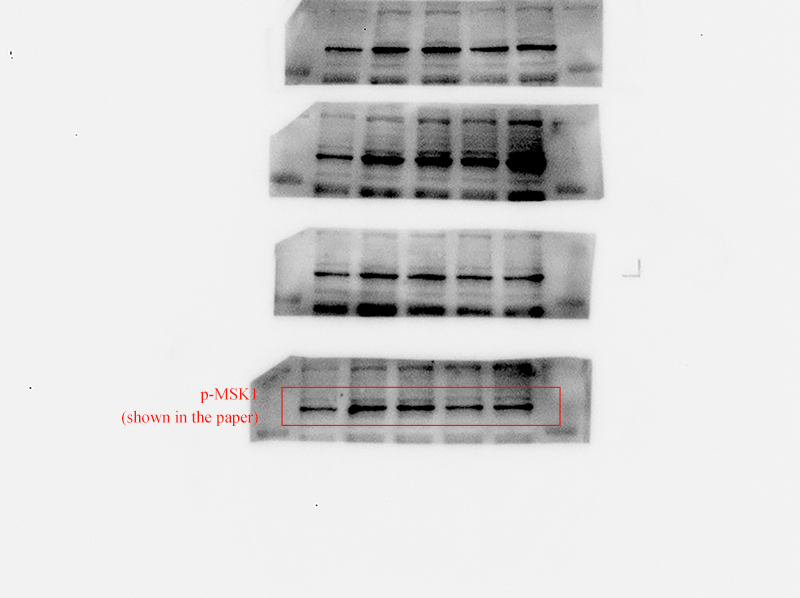

Supplement: Supplementary file 1 [file DataSheet1.ZIP › Original western blot-cells/p-MSK-B.tif]

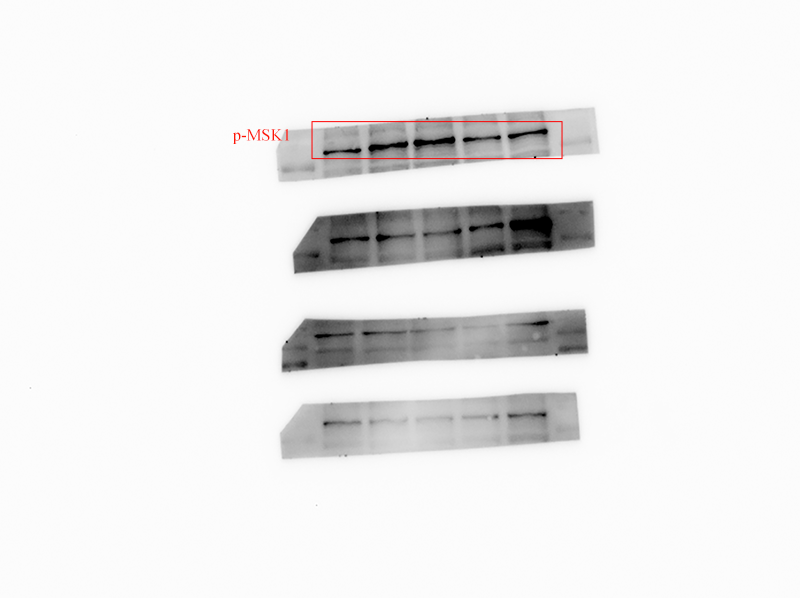

Supplement: Supplementary file 1 [file DataSheet1.ZIP › Original western blot-cells/p-MSK-C.tif]

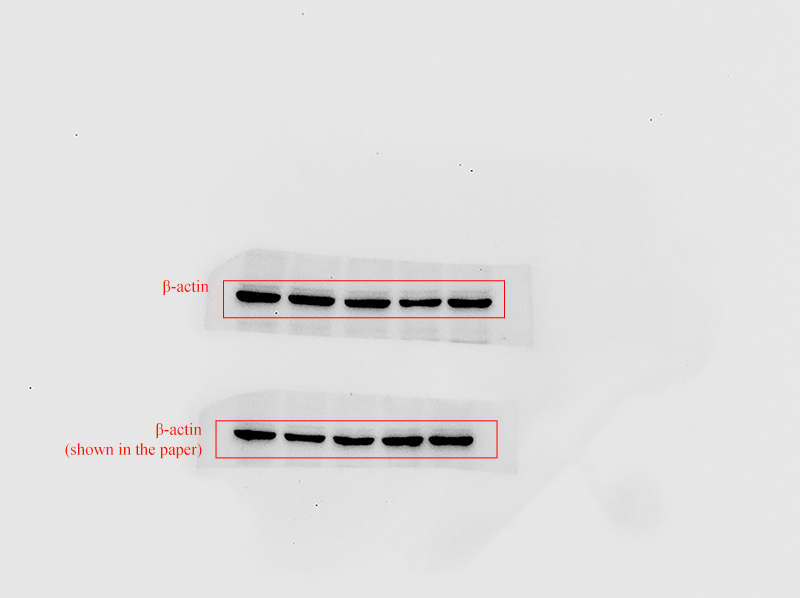

Supplement: Supplementary file 1 [file DataSheet1.ZIP › Original western blot-cells/a┬-actin-A.tif]

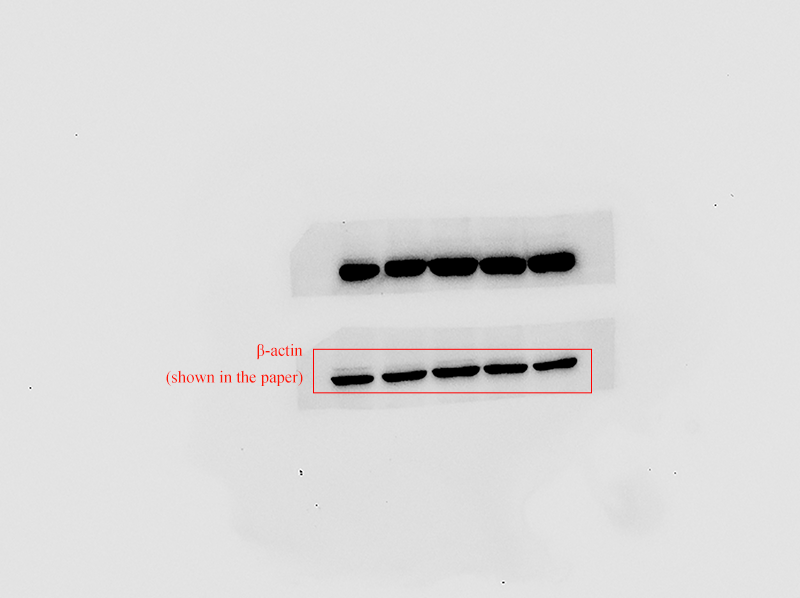

Supplement: Supplementary file 1 [file DataSheet1.ZIP › Original western blot-cells/a┬-actin-B.tif]

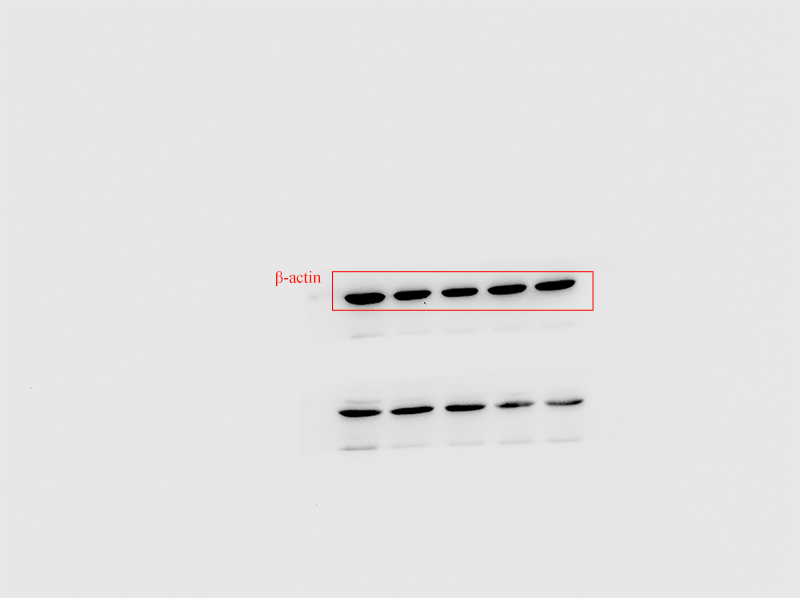

Supplement: Supplementary file 1 [file DataSheet1.ZIP › Original western blot-cells/a┬-actin-C.tif]

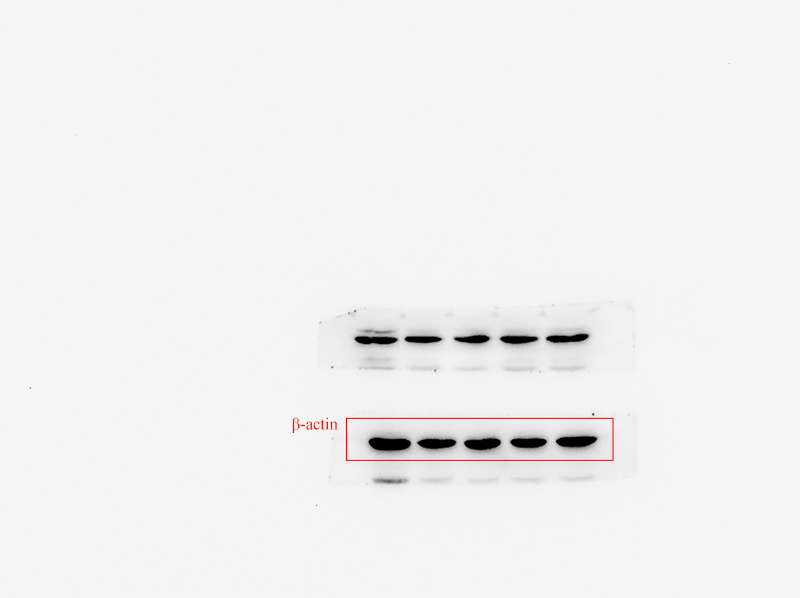

Supplement: Supplementary file 1 [file DataSheet1.ZIP › Original western blot-cells/a┬-actin-D.tif]

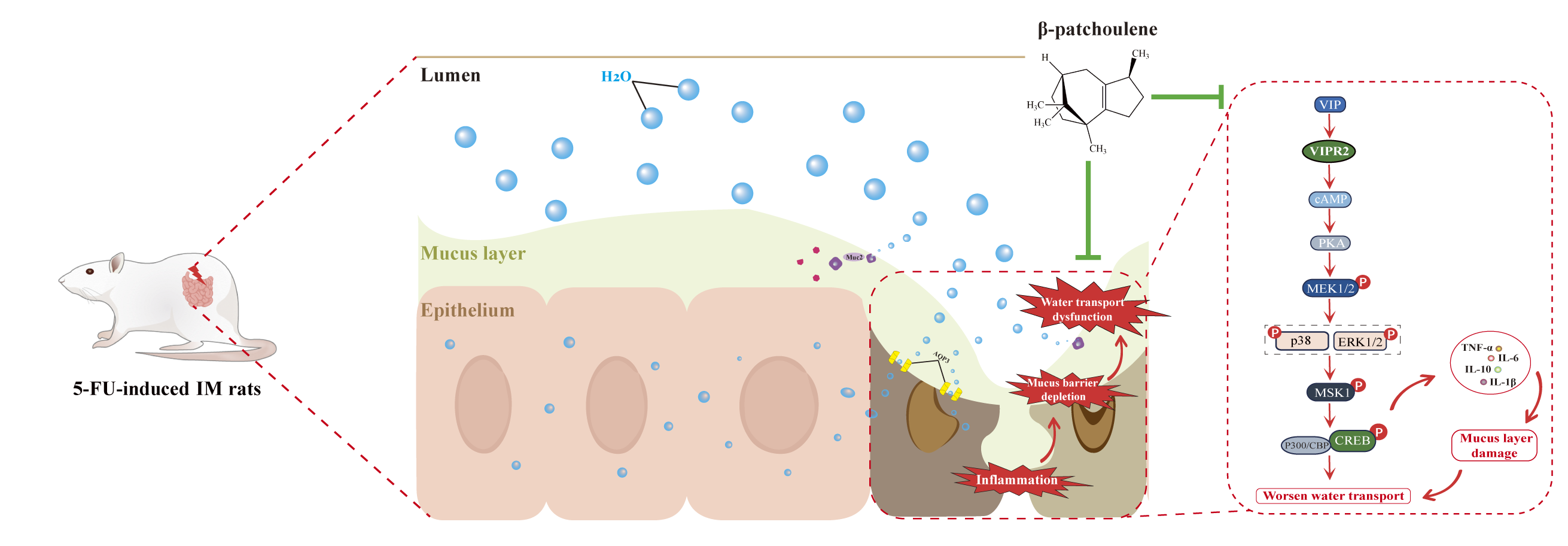

Supplement: Supplementary file 2 [file Image1.TIF]

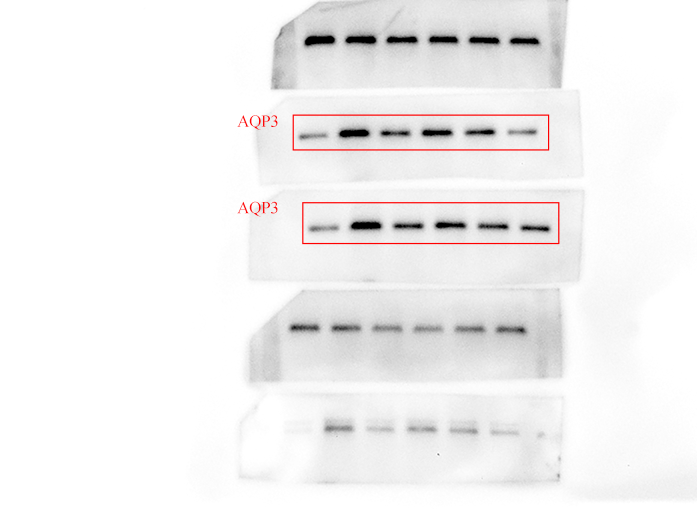

Supplement: Supplementary file 3 [file DataSheet2.ZIP › Original western blot-rats/AQP3-A.tif]

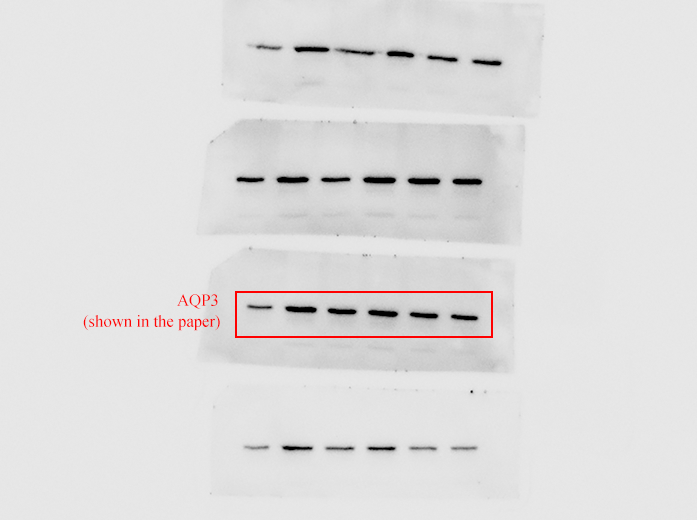

Supplement: Supplementary file 3 [file DataSheet2.ZIP › Original western blot-rats/AQP3-B.tif]

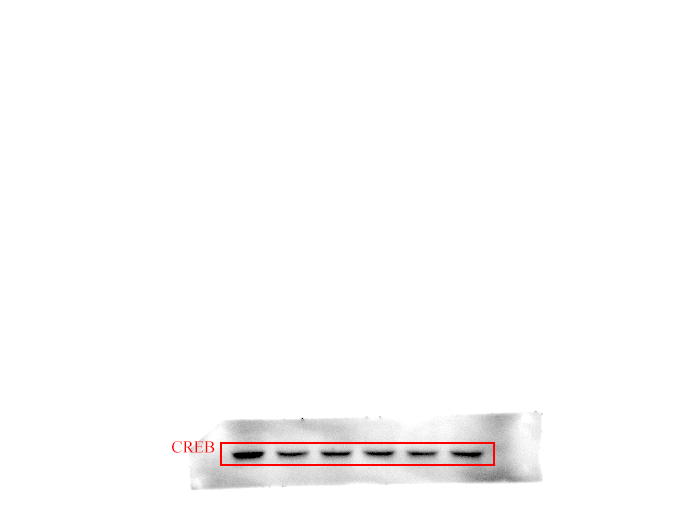

Supplement: Supplementary file 3 [file DataSheet2.ZIP › Original western blot-rats/CREB-A.tif]

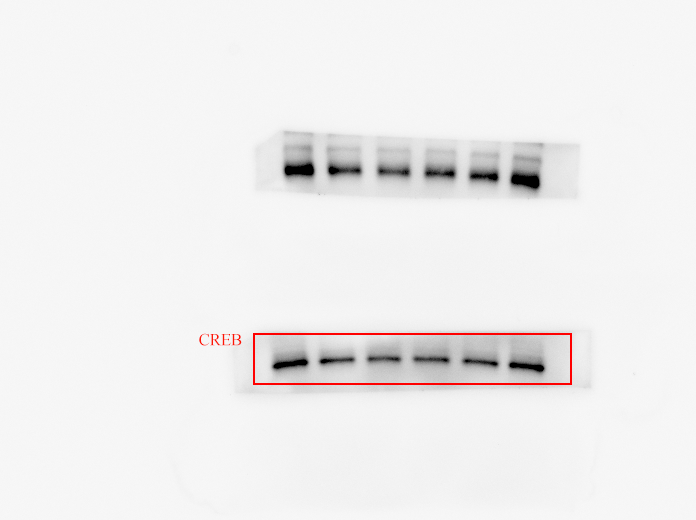

Supplement: Supplementary file 3 [file DataSheet2.ZIP › Original western blot-rats/CREB-B.tif]

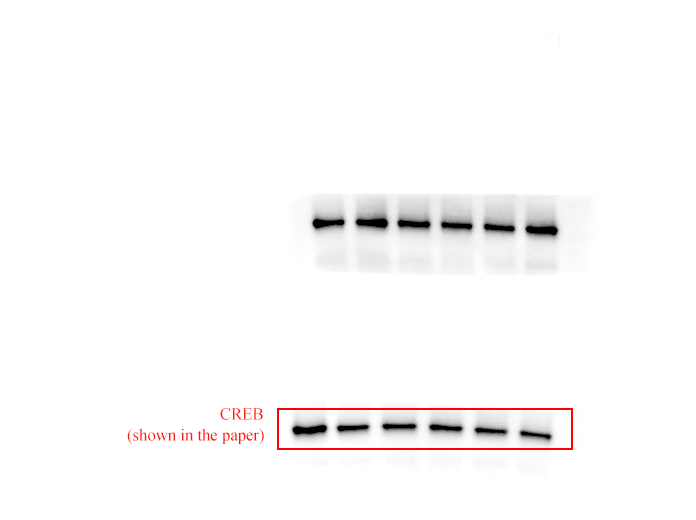

Supplement: Supplementary file 3 [file DataSheet2.ZIP › Original western blot-rats/CREB-C.tif]

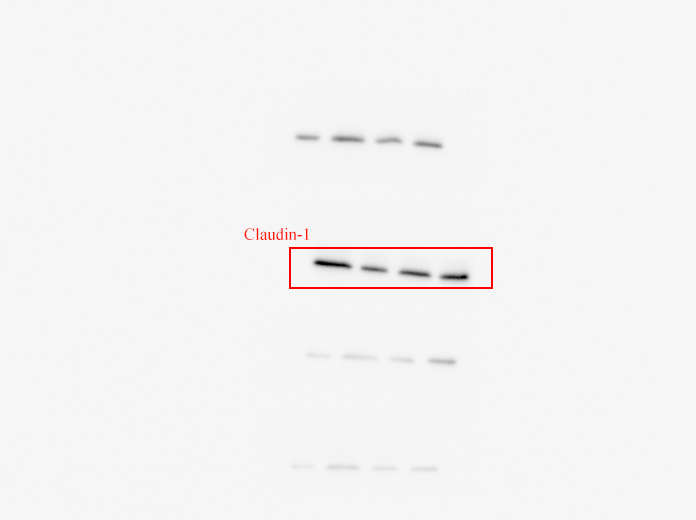

Supplement: Supplementary file 3 [file DataSheet2.ZIP › Original western blot-rats/Claudin-1-A.tif]

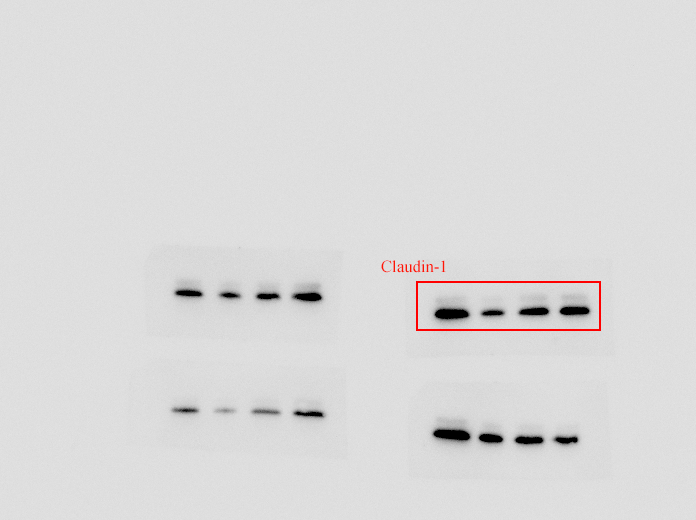

Supplement: Supplementary file 3 [file DataSheet2.ZIP › Original western blot-rats/Claudin-1-B.tif]

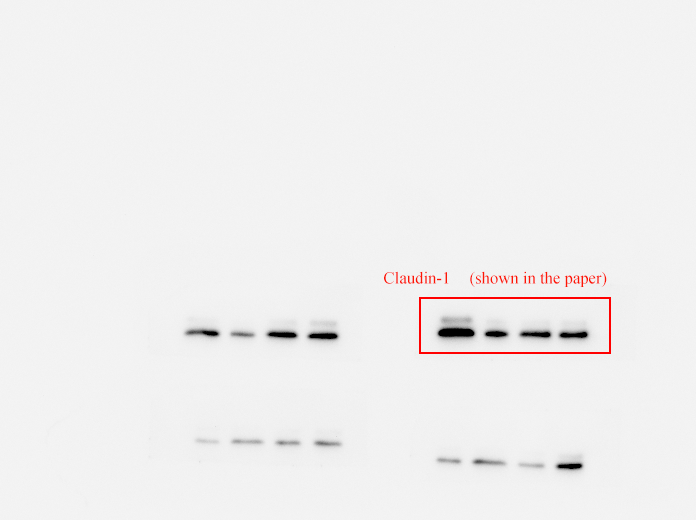

Supplement: Supplementary file 3 [file DataSheet2.ZIP › Original western blot-rats/Claudin-1-C.tif]

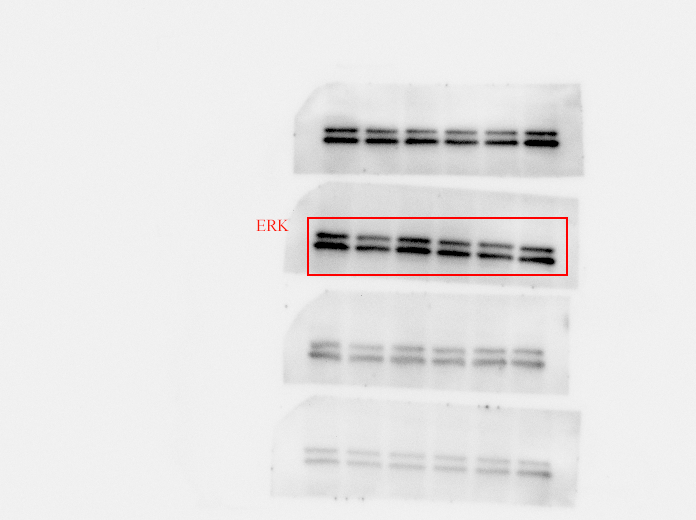

Supplement: Supplementary file 3 [file DataSheet2.ZIP › Original western blot-rats/ERK-A.tif]

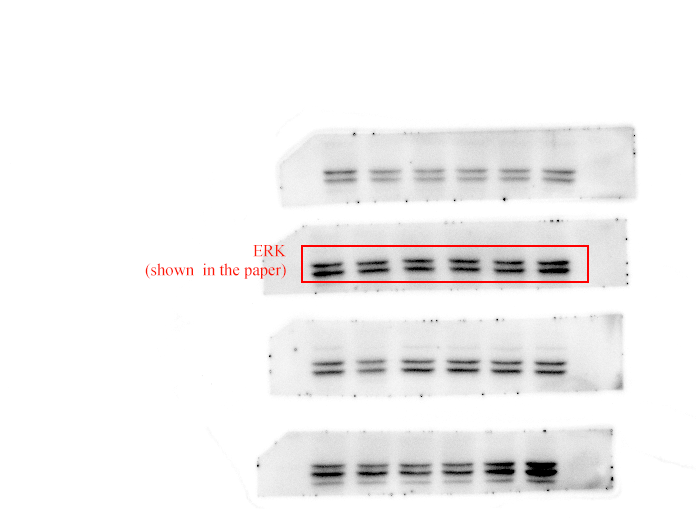

Supplement: Supplementary file 3 [file DataSheet2.ZIP › Original western blot-rats/ERK-B.tif]

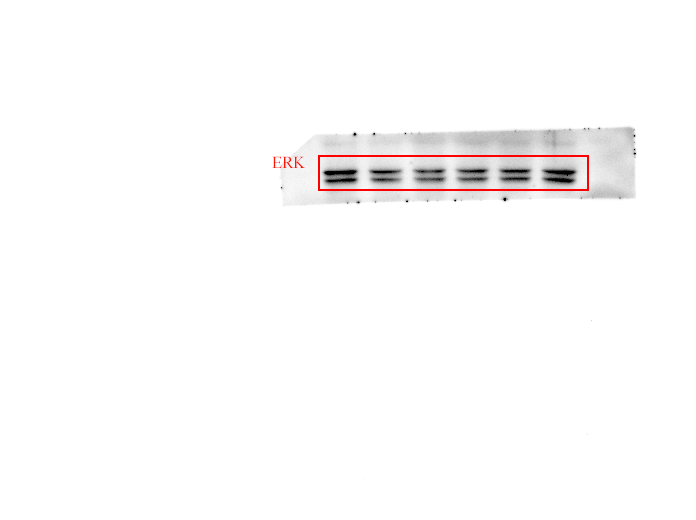

Supplement: Supplementary file 3 [file DataSheet2.ZIP › Original western blot-rats/ERK-C.tif]

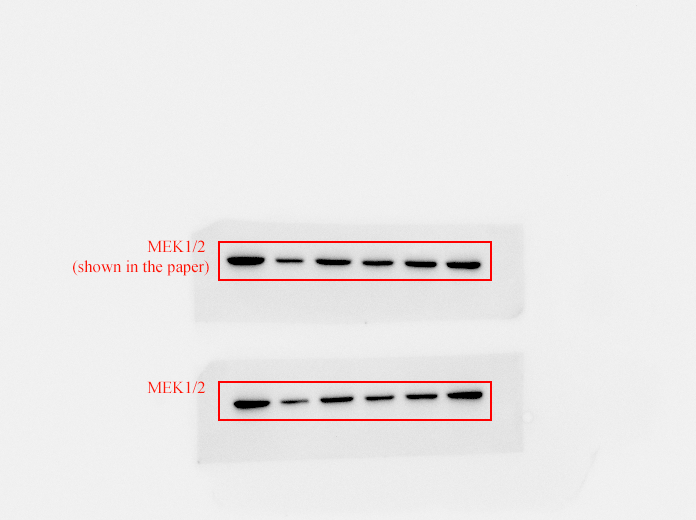

Supplement: Supplementary file 3 [file DataSheet2.ZIP › Original western blot-rats/MEK-A.tif]

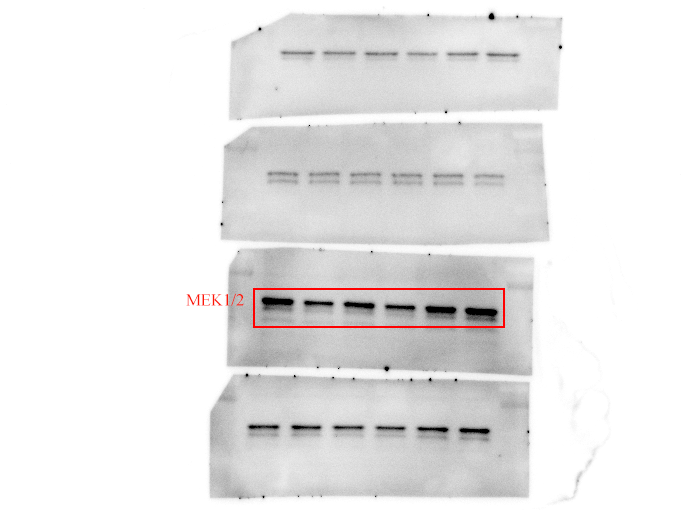

Supplement: Supplementary file 3 [file DataSheet2.ZIP › Original western blot-rats/MEK-B.tif]

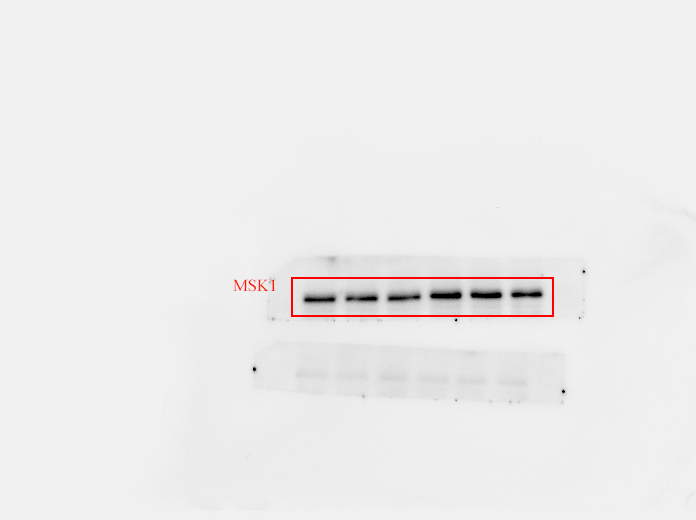

Supplement: Supplementary file 3 [file DataSheet2.ZIP › Original western blot-rats/MSK1-A.tif]

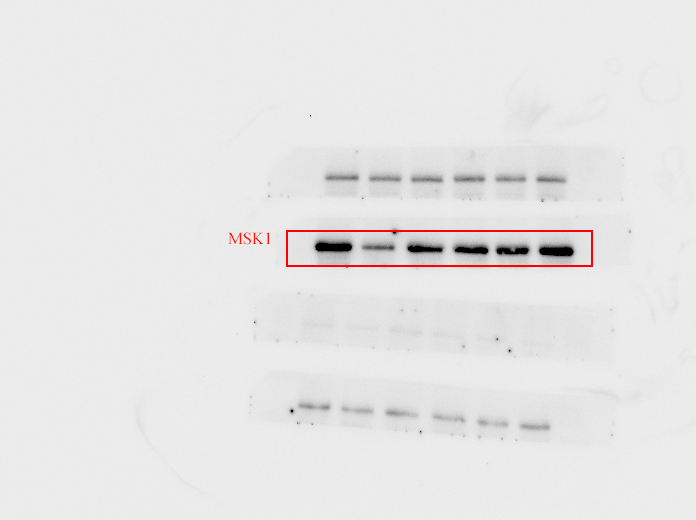

Supplement: Supplementary file 3 [file DataSheet2.ZIP › Original western blot-rats/MSK1-B.tif]

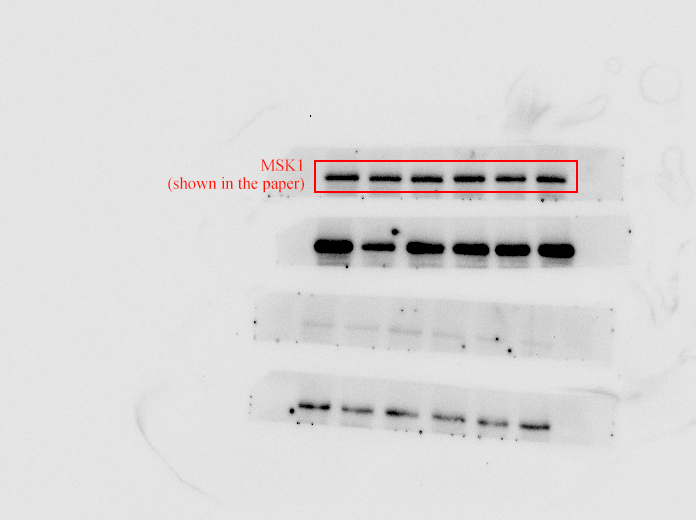

Supplement: Supplementary file 3 [file DataSheet2.ZIP › Original western blot-rats/MSK1-C.tif]

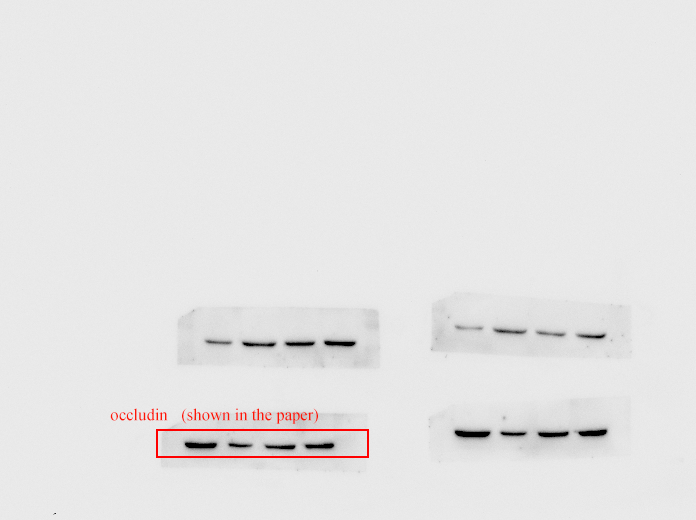

Supplement: Supplementary file 3 [file DataSheet2.ZIP › Original western blot-rats/Occludin-A.tif]

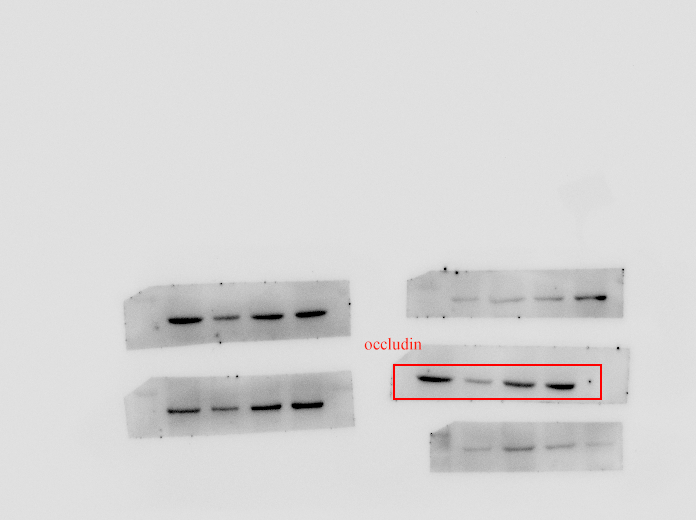

Supplement: Supplementary file 3 [file DataSheet2.ZIP › Original western blot-rats/Occludin-B.tif]

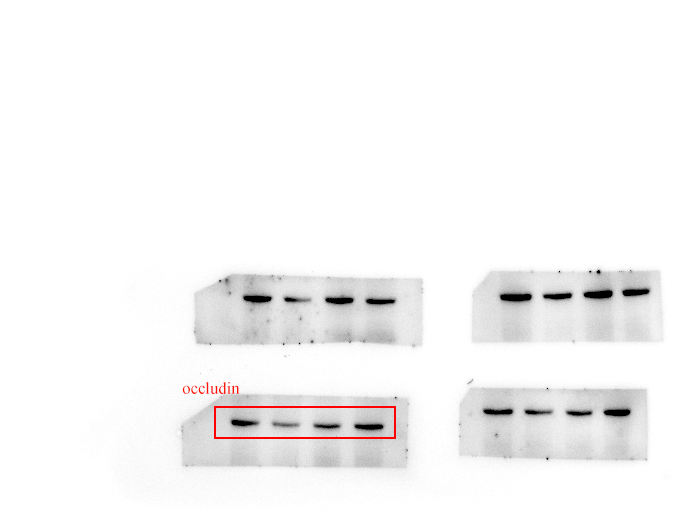

Supplement: Supplementary file 3 [file DataSheet2.ZIP › Original western blot-rats/Occludin-C.tif]

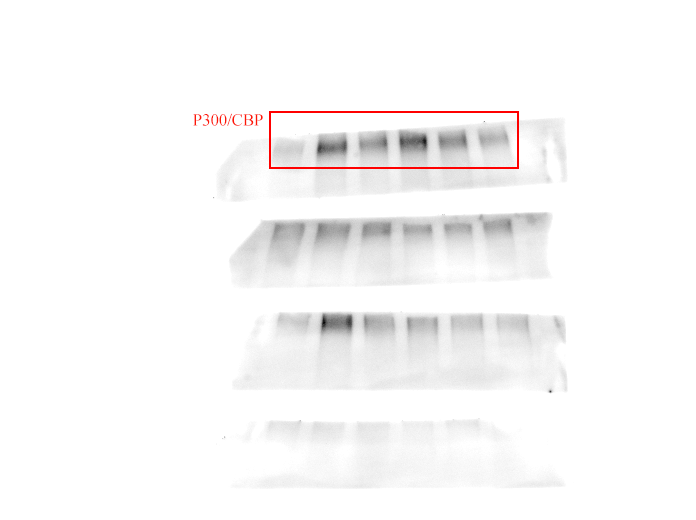

Supplement: Supplementary file 3 [file DataSheet2.ZIP › Original western blot-rats/P300 CBP-A.tif]

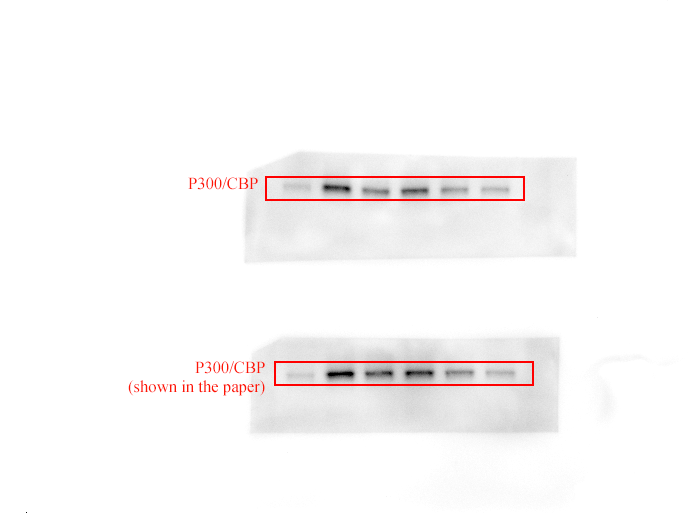

Supplement: Supplementary file 3 [file DataSheet2.ZIP › Original western blot-rats/P300 CBP-B.tif]

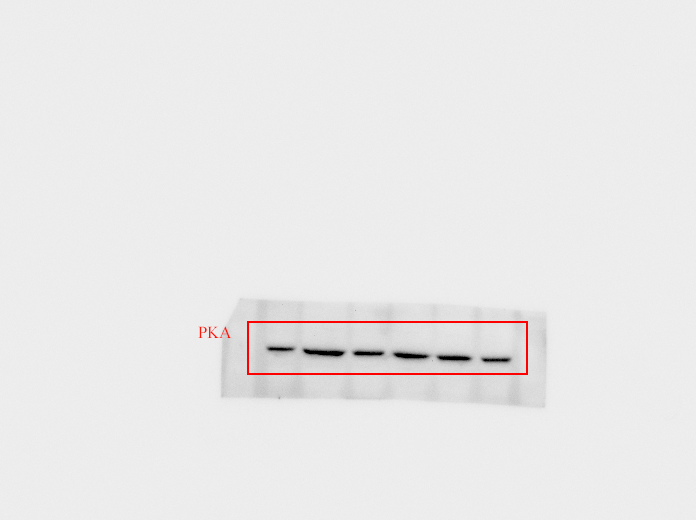

Supplement: Supplementary file 3 [file DataSheet2.ZIP › Original western blot-rats/PKA-A.tif]

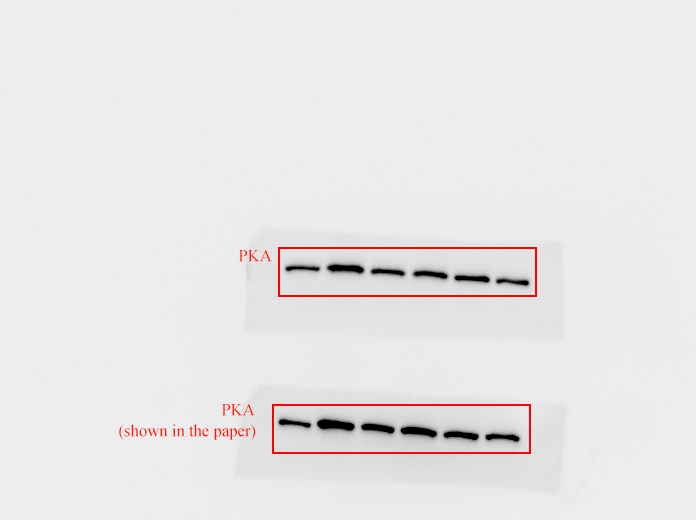

Supplement: Supplementary file 3 [file DataSheet2.ZIP › Original western blot-rats/PKA-B.tif]

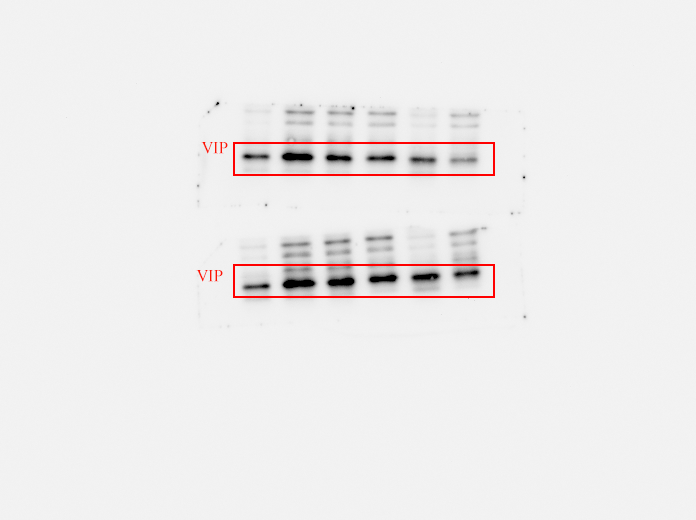

Supplement: Supplementary file 3 [file DataSheet2.ZIP › Original western blot-rats/VIP-A.tif]

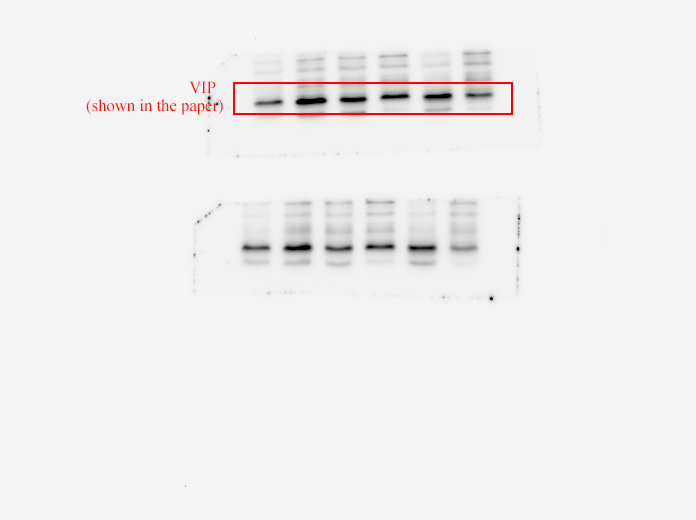

Supplement: Supplementary file 3 [file DataSheet2.ZIP › Original western blot-rats/VIP-B.tif]

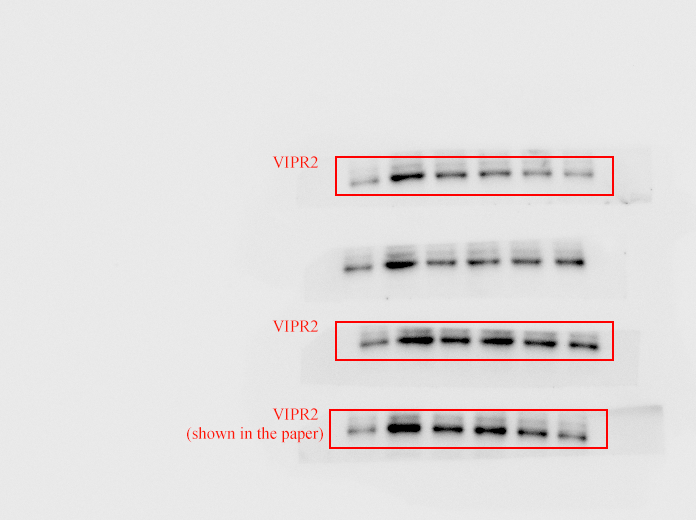

Supplement: Supplementary file 3 [file DataSheet2.ZIP › Original western blot-rats/VIPR2.tif]

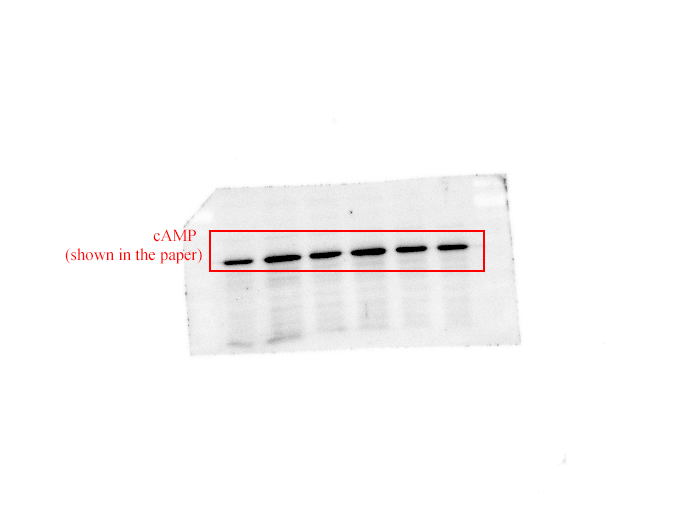

Supplement: Supplementary file 3 [file DataSheet2.ZIP › Original western blot-rats/cAMP-A.tif]

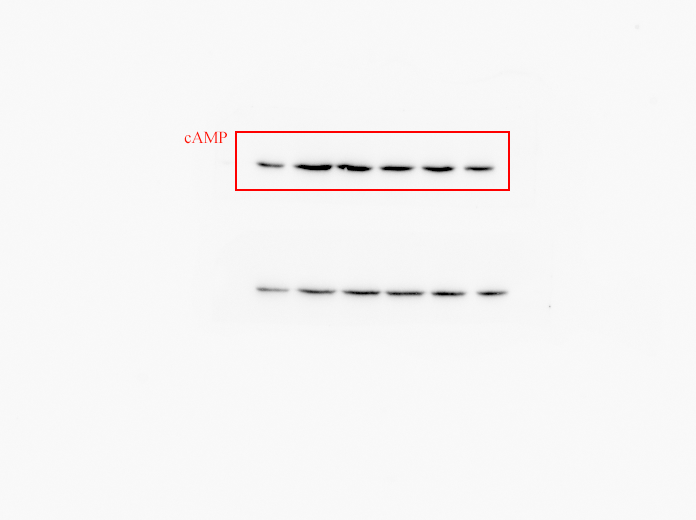

Supplement: Supplementary file 3 [file DataSheet2.ZIP › Original western blot-rats/cAMP-B.tif]

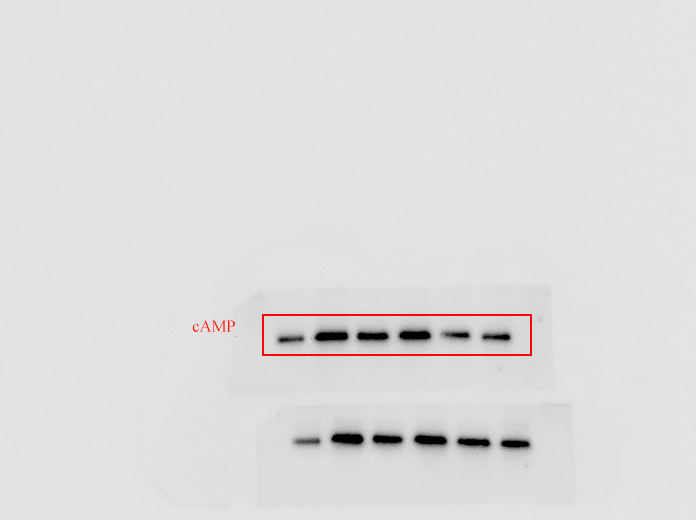

Supplement: Supplementary file 3 [file DataSheet2.ZIP › Original western blot-rats/cAMP-C.tif]

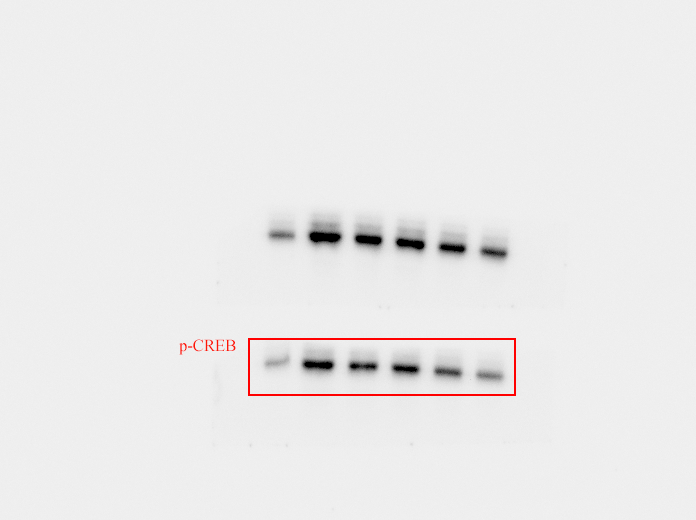

Supplement: Supplementary file 3 [file DataSheet2.ZIP › Original western blot-rats/p-CREB-A.tif]

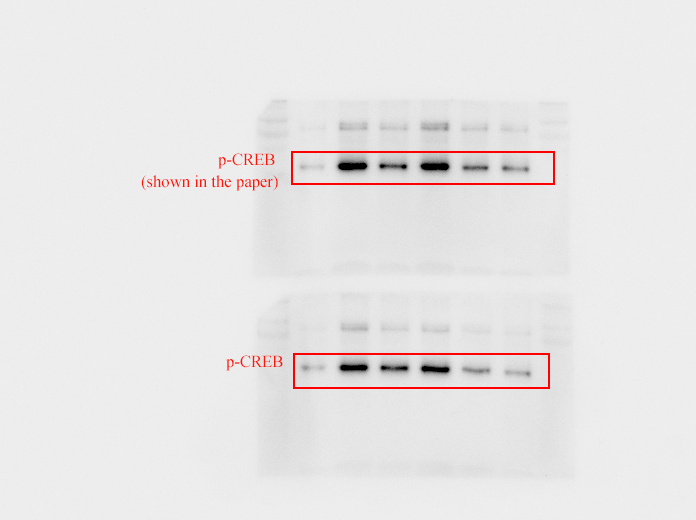

Supplement: Supplementary file 3 [file DataSheet2.ZIP › Original western blot-rats/p-CREB-B.tif]

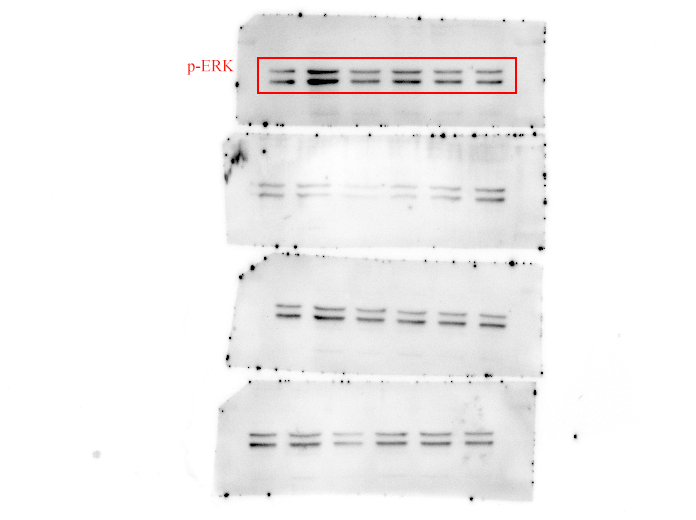

Supplement: Supplementary file 3 [file DataSheet2.ZIP › Original western blot-rats/p-ERK-A.tif]

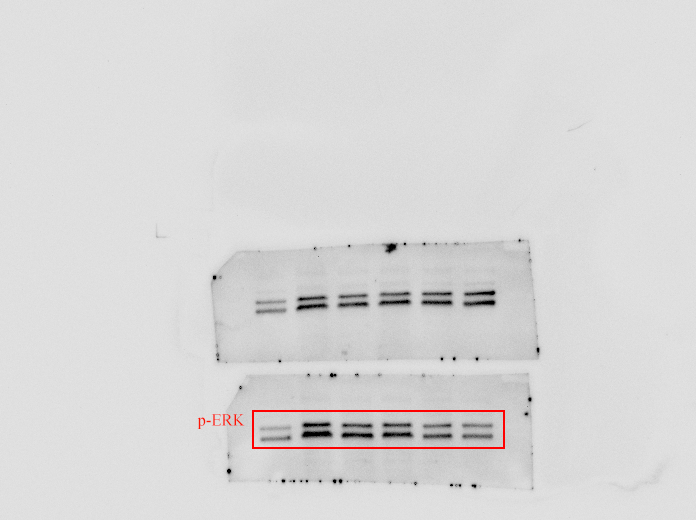

Supplement: Supplementary file 3 [file DataSheet2.ZIP › Original western blot-rats/p-ERK-B.tif]

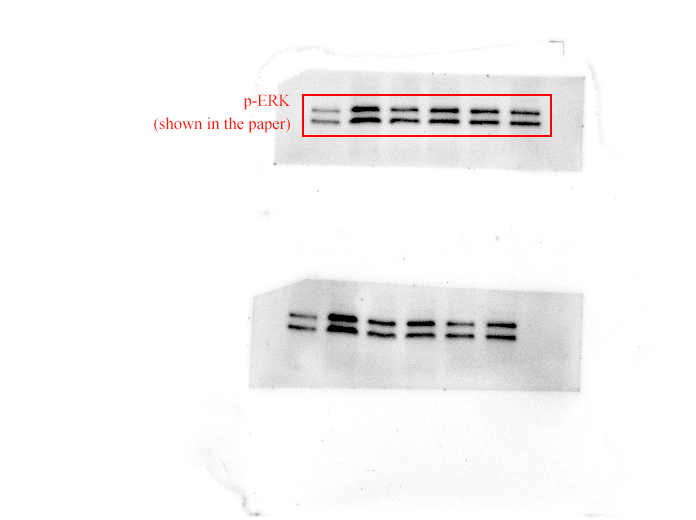

Supplement: Supplementary file 3 [file DataSheet2.ZIP › Original western blot-rats/p-ERK-C.tif]

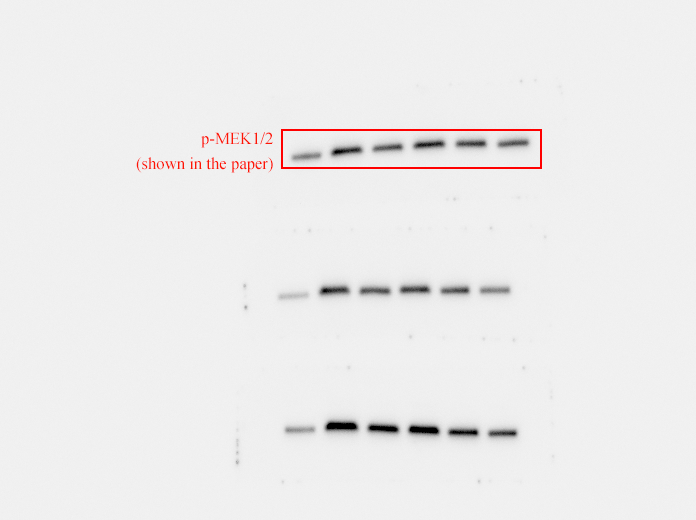

Supplement: Supplementary file 3 [file DataSheet2.ZIP › Original western blot-rats/p-MEK-A.tif]

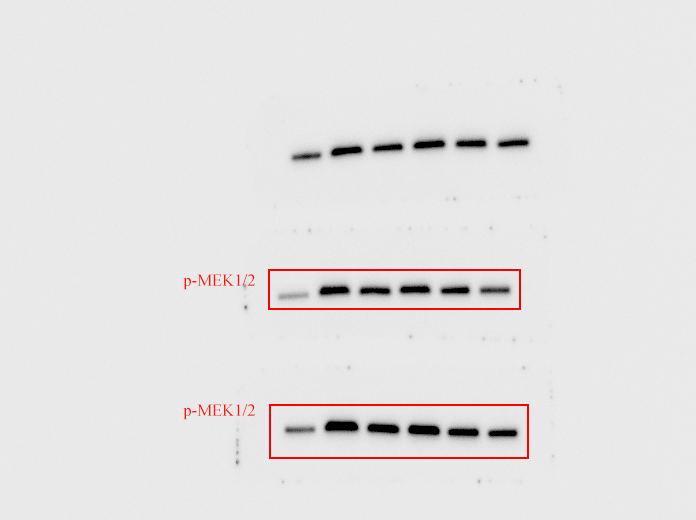

Supplement: Supplementary file 3 [file DataSheet2.ZIP › Original western blot-rats/p-MEK-B.tif]

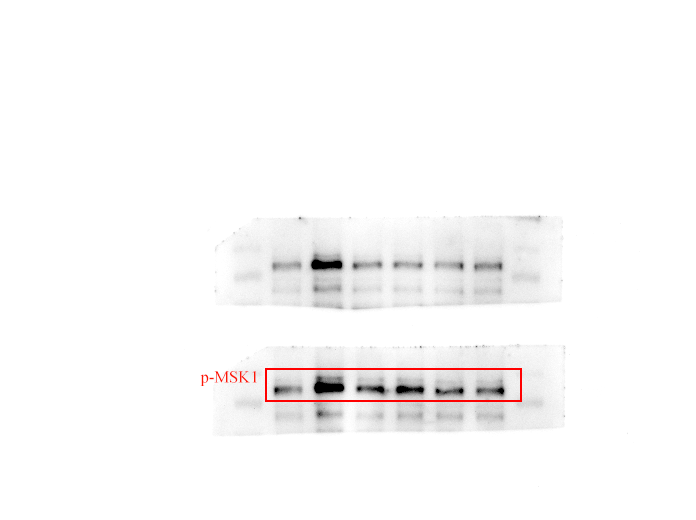

Supplement: Supplementary file 3 [file DataSheet2.ZIP › Original western blot-rats/p-MSK-B.tif]

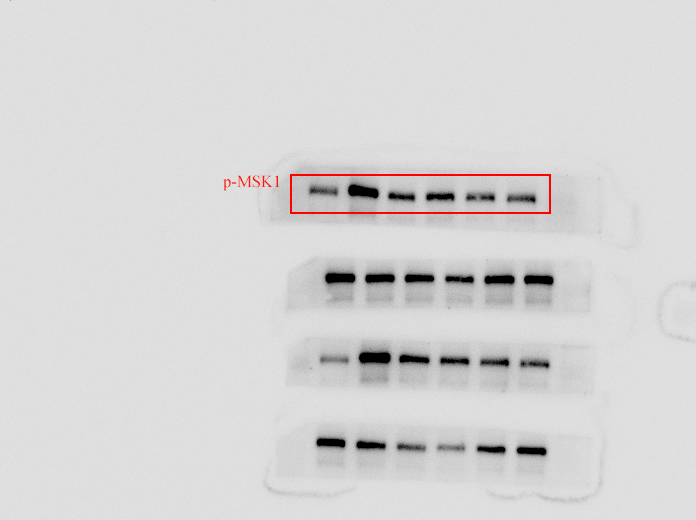

Supplement: Supplementary file 3 [file DataSheet2.ZIP › Original western blot-rats/p-MSK-C.tif]

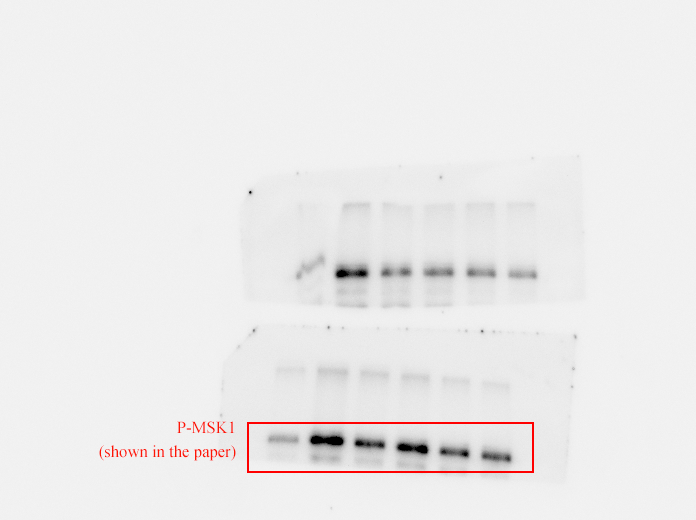

Supplement: Supplementary file 3 [file DataSheet2.ZIP › Original western blot-rats/p-MSK1-A.tif]

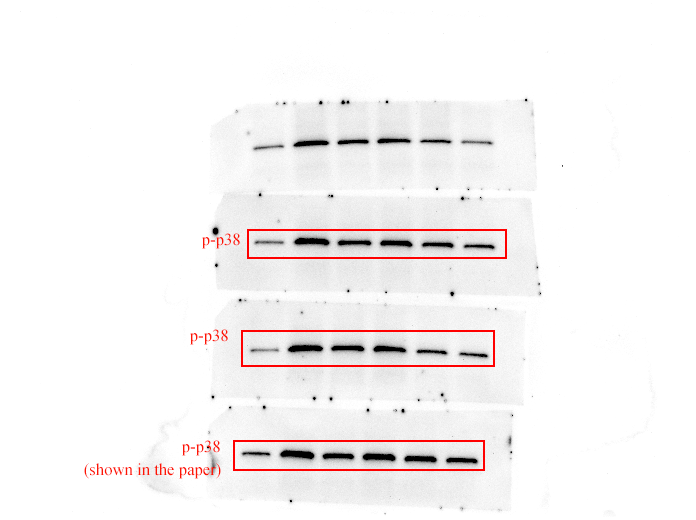

Supplement: Supplementary file 3 [file DataSheet2.ZIP › Original western blot-rats/p-p38.tif]

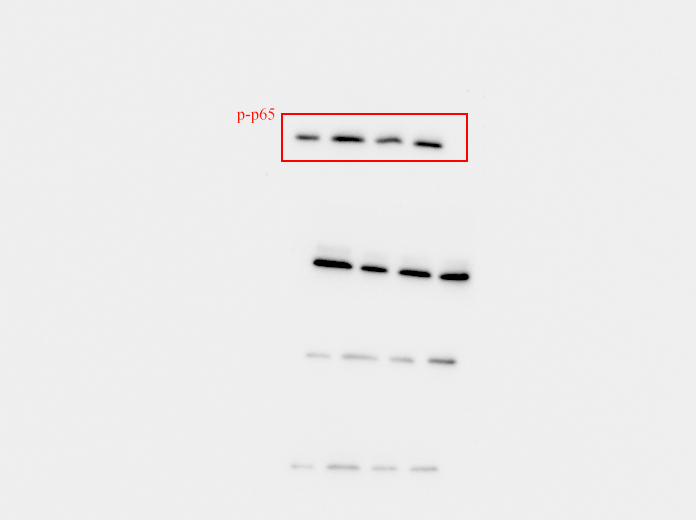

Supplement: Supplementary file 3 [file DataSheet2.ZIP › Original western blot-rats/p-p65-A.tif]

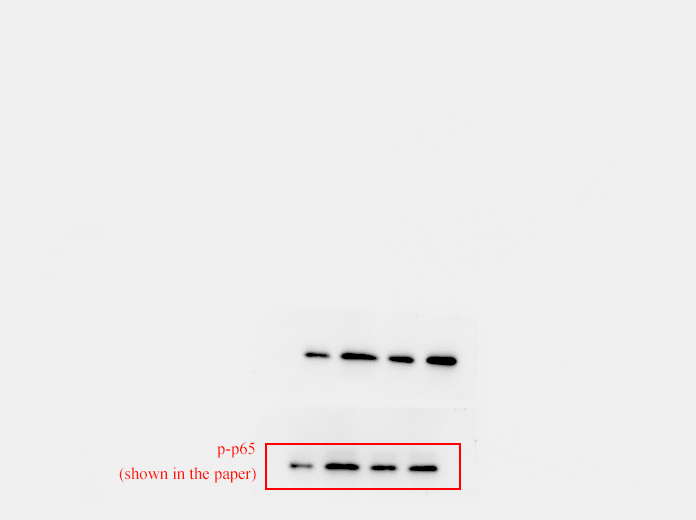

Supplement: Supplementary file 3 [file DataSheet2.ZIP › Original western blot-rats/p-p65-B.tif]

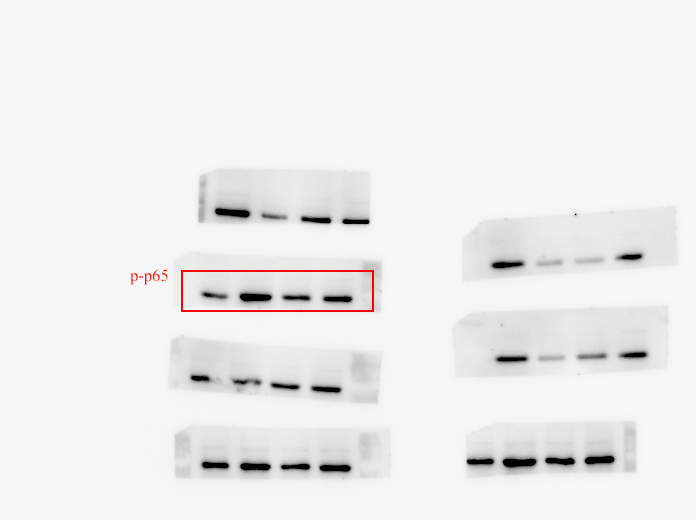

Supplement: Supplementary file 3 [file DataSheet2.ZIP › Original western blot-rats/p-p65-C.tif]

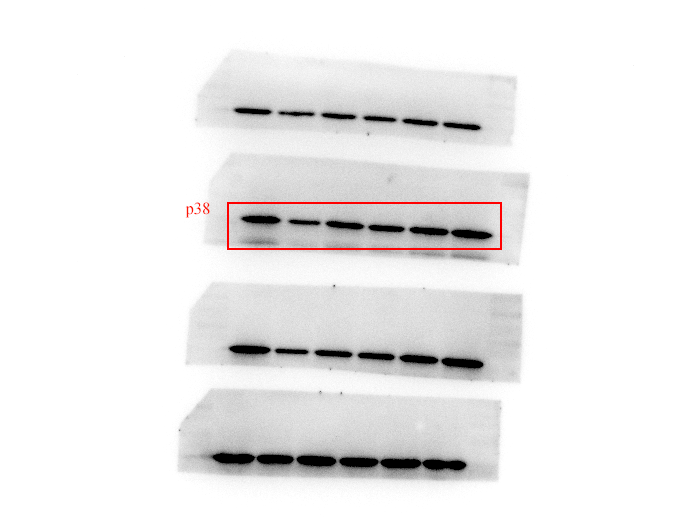

Supplement: Supplementary file 3 [file DataSheet2.ZIP › Original western blot-rats/p38-A.tif]

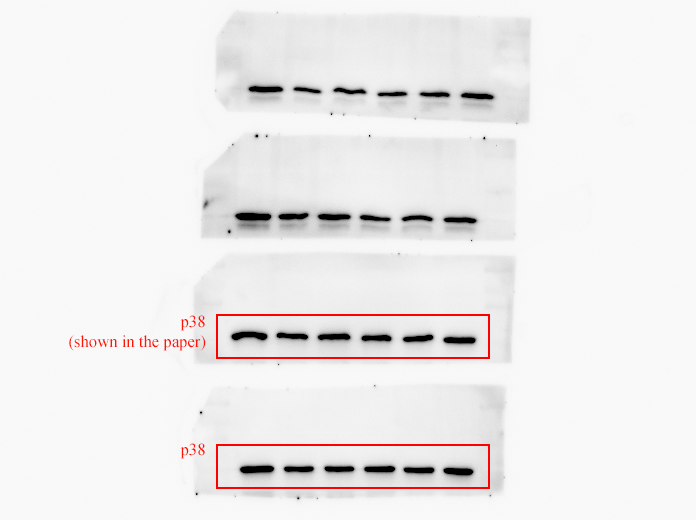

Supplement: Supplementary file 3 [file DataSheet2.ZIP › Original western blot-rats/p38-B.tif]

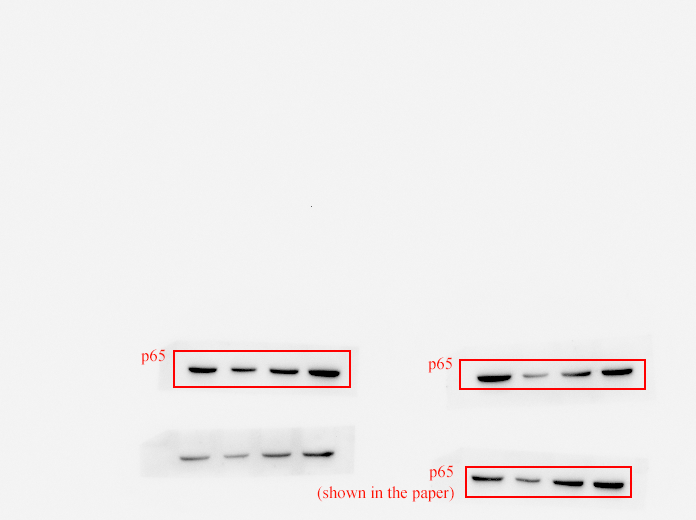

Supplement: Supplementary file 3 [file DataSheet2.ZIP › Original western blot-rats/p65.tif]

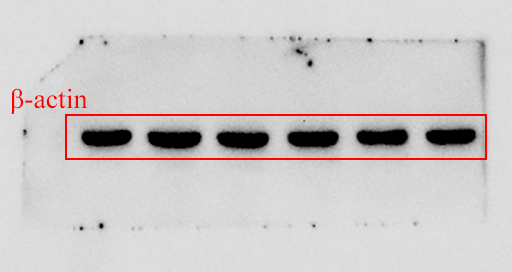

Supplement: Supplementary file 3 [file DataSheet2.ZIP › Original western blot-rats/a┬-actin-A.tif]

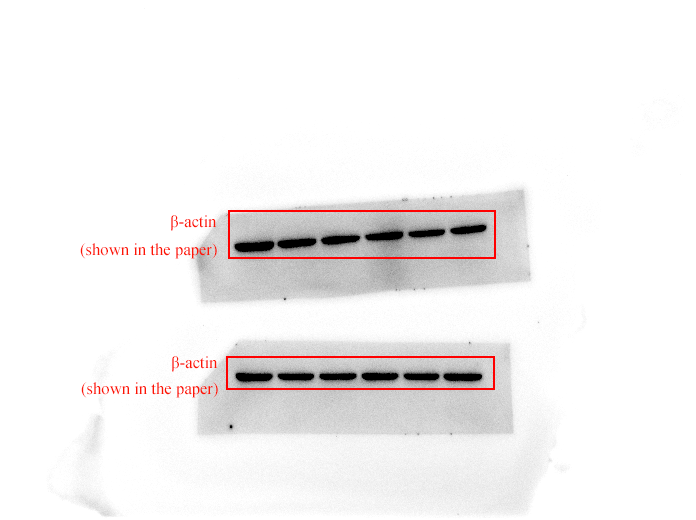

Supplement: Supplementary file 3 [file DataSheet2.ZIP › Original western blot-rats/a┬-actin-B.tif]

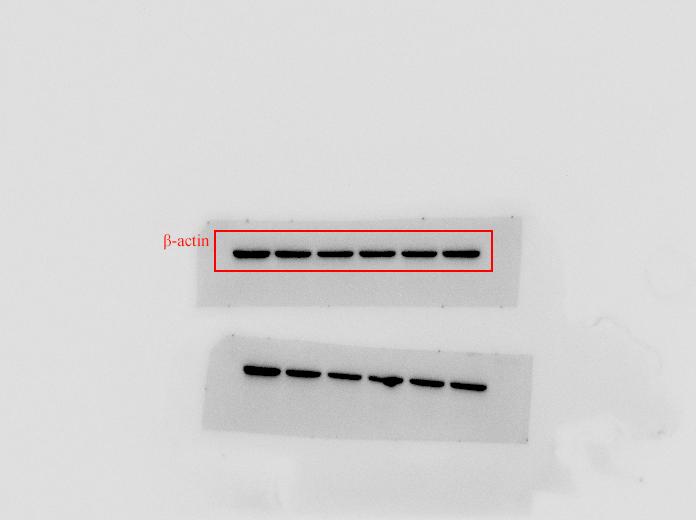

Supplement: Supplementary file 3 [file DataSheet2.ZIP › Original western blot-rats/a┬-actin-C.tif]

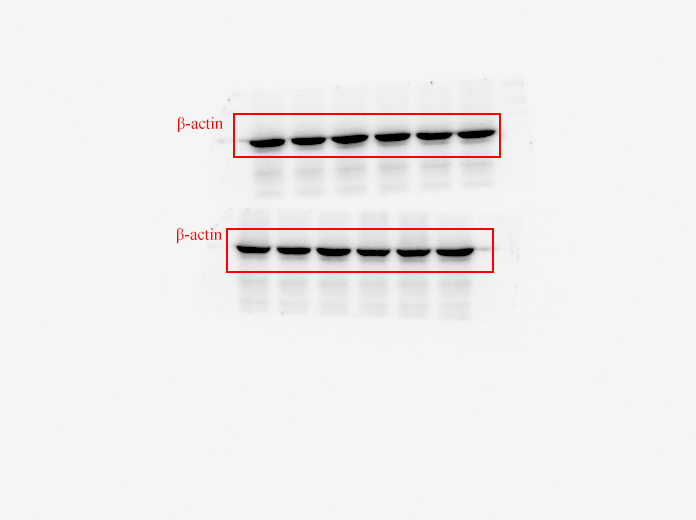

Supplement: Supplementary file 3 [file DataSheet2.ZIP › Original western blot-rats/a┬-actin-D.tif]

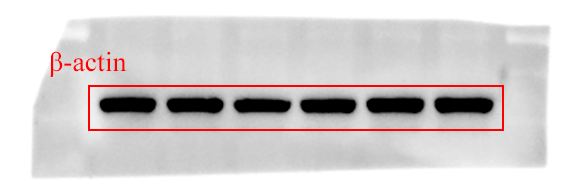

Supplement: Supplementary file 3 [file DataSheet2.ZIP › Original western blot-rats/a┬-actin-E.tif]

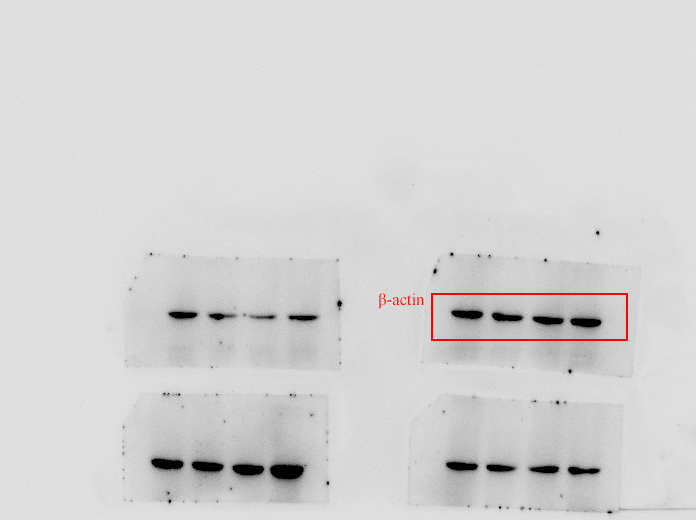

Supplement: Supplementary file 3 [file DataSheet2.ZIP › Original western blot-rats/a┬-actin-F.tif]

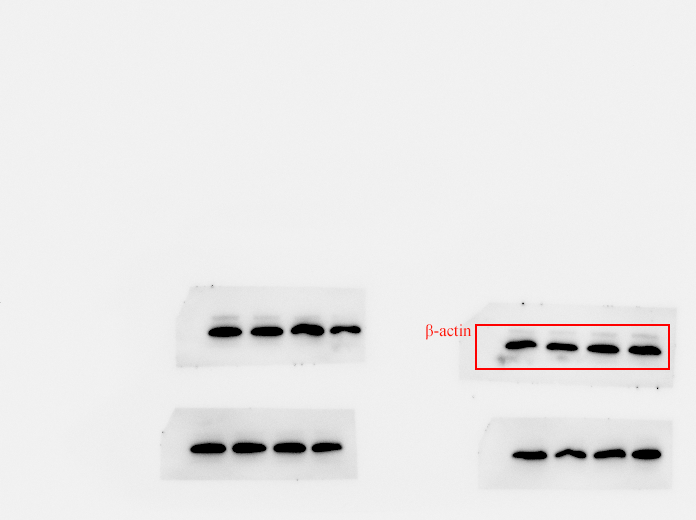

Supplement: Supplementary file 3 [file DataSheet2.ZIP › Original western blot-rats/a┬-actin-G.tif]

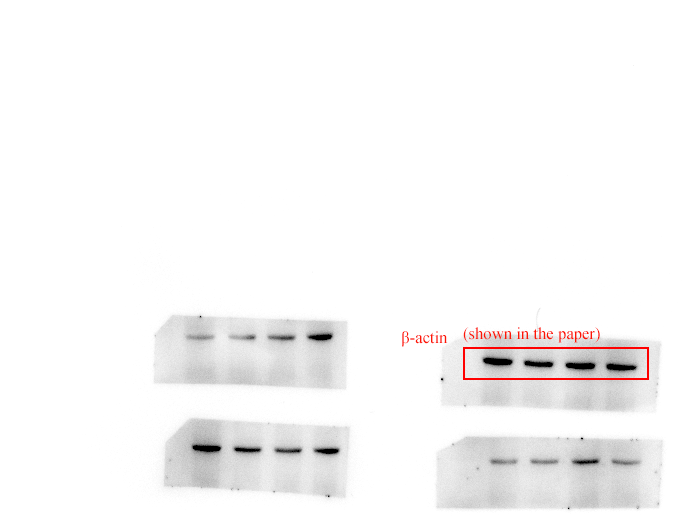

Supplement: Supplementary file 3 [file DataSheet2.ZIP › Original western blot-rats/a┬-actin-H.tif]
